# *N*^*6*^-methyladenosine modification of *CENPK* mRNA by ZC3H13 promotes cervical cancer stemness and chemoresistance

**DOI:** 10.1186/s40779-022-00378-z

**Published:** 2022-04-14

**Authors:** Xian Lin, Feng Wang, Jian Chen, Jing Liu, Yi-Bin Lin, Li Li, Chuan-Ben Chen, Qin Xu

**Affiliations:** 1 Departments of Gynecology, Fujian Cancer Hospital and Fujian Medical University Cancer Hospital, Fujian Medical University, Fuzhou, 350014 China; 2 Department of Radiation Oncology, Fujian Cancer Hospital and Fujian Medical University Cancer Hospital, Fujian Medical University, Fuzhou, 350014 China; 3 Shenzhen Key Laboratory of Immunity and Inflammatory Diseases, Peking University Shenzhen Hospital, Shenzhen Peking University-the Hong Kong University of Science and Technology Medical Center, Shenzhen, 518036 Guangdong China; 4 Outpatient Department, Fujian Hospital of People’s Armed Police, Fujian Medical University, Fuzhou, 350014 China

**Keywords:** *N*^*6*^-methyladenosine, Centromere protein K, Cervical cancer, Stemness, Chemoresistance

## Abstract

**Background:**

Stemness and chemoresistance contribute to cervical cancer recurrence and metastasis. In the current study, we determined the relevant players and role of *N*^*6*^-methyladenine (m^6^A) RNA methylation in cervical cancer progression.

**Methods:**

The roles of m^6^A RNA methylation and centromere protein K (CENPK) in cervical cancer were analyzed using bioinformatics analysis. Methylated RNA immunoprecipitation was adopted to detect m^6^A modification of *CENPK* mRNA. Human cervical cancer clinical samples, cell lines, and xenografts were used for analyzing gene expression and function. Immunofluorescence staining and the tumorsphere formation, clonogenic, MTT, and EdU assays were performed to determine cell stemness, chemoresistance, migration, invasion, and proliferation in HeLa and SiHa cells, respectively. Western blot analysis, co-immunoprecipitation, chromatin immunoprecipitation, and luciferase reporter, cycloheximide chase, and cell fractionation assays were performed to elucidate the underlying mechanism.

**Results:**

Bioinformatics analysis of public cancer datasets revealed firm links between m^6^A modification patterns and cervical cancer prognosis, especially through ZC3H13-mediated m^6^A modification of *CENPK* mRNA. CENPK expression was elevated in cervical cancer, associated with cancer recurrence, and independently predicts poor patient prognosis [hazard ratio = 1.413, 95% confidence interval = 1.078 − 1.853, *P* = 0.012]. Silencing of *CENPK* prolonged the overall survival time of cervical cancer-bearing mice and improved the response of cervical cancer tumors to chemotherapy in vivo (*P* < 0.001). We also showed that CENPK was directly bound to SOX6 and disrupted the interactions of CENPK with β-catenin, which promoted β-catenin expression and nuclear translocation, facilitated p53 ubiquitination, and led to activation of Wnt/β-catenin signaling, but suppression of the p53 pathway. This dysregulation ultimately enhanced the tumorigenic pathways required for cell stemness, DNA damage repair pathways necessary for cisplatin/carboplatin resistance, epithelial-mesenchymal transition involved in metastasis, and DNA replication that drove tumor cell proliferation.

**Conclusions:**

CENPK was shown to have an oncogenic role in cervical cancer and can thus serve as a prognostic indicator and novel target for cervical cancer treatment.

**Supplementary Information:**

The online version contains supplementary material available at 10.1186/s40779-022-00378-z.

## Background

Existing data have shown that cervical cancer ranks as the second leading cause of cancer-associated deaths among women 20–39 years of age [1, 2], with treatment failure and poor prognosis attributed to cervical cancer cell stemness, chemoresistance, and metastasis [3]. Identifying alternative targets therefore represents an urgent priority to best utilize these tumorigenic properties in cervical cancer.

Several recent studies have suggested that *N*^*6*^-methyladenine (m^6^A) RNA methylation, the most abundant RNA modification that functionally affects mRNAs, lncRNAs, and miRNAs, is also involved in cervical cancer progression [4–6]. Many proteins are known to contribute to the m^6^A RNA methylation process; specifically, ZC3H13, METTL3, WTAP, and others function as methyltransferases to catalyze the addition of m^6^A marks on RNA. In addition to methyltransferases, demethylases (e.g., FTO and ALKBH5) catalyze the removal of m^6^A marks from RNA, while m^6^A readers, such as YTHDF1/2/3, YTHDC1/2, IGF2BP1/2/3, and others bind RNA to recognize m^6^A marks [7, 8]. The biological roles in modulating cervical cancer progression are poorly understood for many m^6^A-related genes because of the effects in aberrant regulation of downstream signaling pathways.

Previous studies have suggested links between centromere protein K (CENPK) and the progression of malignant tumors, such as ovarian cancer, breast cancer, hepatocellular carcinoma, bladder cancer, oligodendrogliomas, and lung adenocarcinoma [9–14]. CENPK is a subunit of the centromeric complex involved in assembly of kinetochore proteins. In coordination with centromere protein A (CENPA) and other centromere components, CENPK is an essential protein in chromosome segregation and mitotic progression [15]. Despite this broad contribution to different cancers, the relationship between CENPK and cervical cancer remains unknown, and the mechanism by which CENPK dysregulates cancer progression has not been established.

The purpose of the current study was to establish a connection between specific m^6^A methylation patterns and cervical cancer survival rates and prognosis, and to determine the effect of relevant factors on cervical cancer cell stemness, chemoresistance, metastasis, and proliferation using cervical cancer cell lines, animal models, and clinical samples, as well as publicly available cancer datasets. In particular, we focused on the mechanism by which hypermethylation of *CENPK* mRNA results in upregulation, leading to disruption of SOX6 interactions and dysregulation of downstream Wnt and p53 pathway targets. Herein, we proposed a novel regulatory axis that promoted the tumorigenic properties of cervical cancer cells arising from the m^6^A methyltransferase, ZC3H13, through CENPK/SOX6 to the Wnt/β-catenin and p53 signaling pathways.

## Methods

### Cell cultures

Cervical cancer cell lines (HeLa and SiHa) were purchased from the Chinese Academy of Sciences Cell Bank (Shanghai, China) and cultivated in Dulbecco’s modified Eagle medium (DMEM) containing 10% fetal bovine serum. Cervical cancer cells were grown at 37 ℃ with 5% CO_2_ and 95% room air. Cells were routinely measured for *Mycoplasma* contamination.

### Cell transfections

Lentiviral particles harboring shRNA targeting *CENPK* (sh-CENPK) and negative control shRNA (sh-NC) were designed and constructed by GeneChem Corporation (Shanghai, China). Lentivirus particles were transfected into cervical cancer cells using a polybrene reagent. Plasmids (ov-β-catenin and ov-CENPK) were designed and constructed by Vigene Biosciences Corporation (Shandong, China). *CENPK* siRNA (si-CENPK), *SOX6* siRNA (si-SOX6), *p53* siRNA (si-p53), and *ZC3H13* siRNA (si-ZC3H13) were obtained from RiboBio Corporation (Guangzhou, China) (Additional file 1: Table S1). Following the manufacturer’s protocol, plasmids and siRNAs were transduced into cervical cancer cells using Lipofectamine™ 2000 (Invitrogen Corporation, Shanghai, China). After transfection for 48 h-72 h, cells were subjected to further experimentation.

### Tumorsphere formation assay

The protocol for the tumorsphere formation assay was previously described [16]. Briefly, cervical cancer cells (5000 cells/well) were seeded on 6-well ultra-low-attachment plates (Corning, Inc., New York, NY, USA) and cultured in serum-free DMEM/F12 with 2% B27, 20 ng/ml of EGF, and 20 ng/ml of FGF. The tumorspheres were recorded and counted on day 14 post-seeding, and the number and size of tumorspheres were analyzed after passaging for three generations.

### MTT assay

Cervical cancer cells (1000 cells/well) were seeded onto 96-well plates. At the indicated time, MTT (5 mg/ml; Sigma-Aldrich Corporation, St. Louis, MO, USA) was added to each well. After a 4 h incubation, dimethyl sulfoxide (Sigma-Aldrich Corporation) was added to each well. The absorbance value (OD) of each well was detected at 490 nm.

### Colony-formation assay

Cervical cancer cells were plated at a density of 100 cells per well to measure cell proliferation. After a 10 d culture, colonies were fixed with methanol and stained with a hematoxylin solution. The number of colonies (≥ 50 cells) was counted under a light microscope. Cervical cancer cells were treated with cisplatin or carboplatin for 6 h at the indicated concentrations before seeding at a concentration of 500 cells per well to assess cell chemoresistance, as previously described [17]. After a 10 d of incubation, the colonies were fixed, stained, and photographed for analysis.

### Transwell assays

Transwell assays were used to evaluate the migration and invasion capacity of cervical cancer cells. Cells suspension were seeded to the upper chamber of Transwell plates coated with Matrigel (BD Biosciences, Franklin Lakes, NJ, USA) for invasion assays or non-coated for migration assays; DMEM supplemented with 10% FBS was added to the lower chambers. The migrated and invaded cells were fixed, stained, and photographed under light microscopy.

### EdU incorporation assays

EdU assay was conducted using the Apollo567 in vitro imaging kit (RiboBio Corporation, Guangzhou, China) following the manufacturer’s protocol. After a 2 h treatment with EdU (10 μmol/L), cervical cancer cells were fixed with 4% paraformaldehyde, permeabilized with 0.3% Triton X-100, and co-stained with Apollo fluorescent dyes and DAPI (5 μg/ml). The EdU-positive cells were recorded and calculated under fluorescence microscopy.

### Immunofluorescence

Cervical cancer cells were seeded and grown on coverslips. After incubation, the cells were fixed with 4% paraformaldehyde, permeabilized with 0.2% Triton X-100, and incubated with antibodies (Additional file 1: Table S2). The cells were then co-stained with DAPI (0.2 mg/ml) and photographed with a fluorescence confocal microscope.

### Reverse transcription-polymerase chain reaction (RT-PCR) and quantitative real-time PCR (qPCR)

Total RNAs were extracted from cervical cancer cells or xenografts using TRIzol (Invitrogen Corporation), and cDNAs were generated using a reverse transcription reagent kit (TaKaRa Corporation, Dalian, China). The synthesized cDNAs were adopted as templates for RT-PCR and qPCR with specific primers on Bio-Rad T100 and Bio-Rad CFX 96 (Bio-Rad, Hercules, CA, USA), respectively. β-actin was used as a control. The images were visualized by Bio-Rad GelDoc XR^+^ for RT-PCR. Relative mRNA expression was quantified using the 2^−ΔΔCt^ method for qPCR.

### Western blotting

Cervical cancer cells were lysed in lysis buffer, and proteins were quantified using a BCA protein assay kit (Thermo Scientific, Waltham, MA, USA). After loading, proteins were separated, transferred, and immunoprobed with specific antibodies. Antibodies against the following proteins were used: Flag; CENPK; ZC3H13; SOX6; β-catenin; c-Jun; c-Myc; CCND1; p53; p21; Vimentin; Ubiquitin; GAPDH; Histone; and β-actin. Detailed antibody information is shown in Additional file 1: Table S2. Chemiluminescence was used for protein detection, and the Bio-Rad ChemiDocTM CRS + Molecular Imager was adopted for capturing images.

### Animal studies

The Institutional Animal Ethical Committee, Experimental Animal Center of Fujian Medical University and Fujian Cancer Hospital approved the protocols for animal studies (K2020-036–01). All experiments conformed to all relevant regulatory standards. BALB/c-nu mice were grouped as follows to detect tumor growth: HeLa-sh-NC [mice inoculated with scramble shRNA-infected control HeLa cells (*n* = 5)] and HeLa-sh-CENPK [mice inoculated with CENPK-targeted shRNA-infected HeLa cells (*n* = 5)]. The mice were subcutaneously inoculated with 5 × 10^6^ cells/100 μl in the flank. The longest and the shortest diameters of the growing tumors were measured every 3 d with a caliper, and the tumor volume (V) was counted by the following equation: V = (the longest diameter × the shortest diameter^2^)/2. The mice were grouped as follows to evaluate the tumor-initiating frequency and were inoculated with a series of 5 × 10^5^, 2 × 10^5^, and 5 × 10^4^ cells subcutaneously: HeLa-sh-NC (*n* = 6); and HeLa-sh-CENPK (*n* = 6). The mice bearing subcutaneous xenograft tumors were grouped as follows to evaluate tumor chemoresistance: HeLa-sh-NC (*n* = 10); HeLa-sh-CENPK (*n* = 10); HeLa-sh-NC + cisplatin [mice inoculated with scramble shRNA-infected control HeLa cells and 3 mg/kg of cisplatin once a week intraperitoneally (ip) for 6 weeks (*n* = 10)] [18, 19]; and HeLa-sh-CENPK + cisplatin [mice inoculated with CENPK-targeted shRNA-infected HeLa cells and ip cisplatin (*n* = 10)]. Survival curves were plotted using Kaplan–Meier analyses. The detection of cervical cancer metastatic potential was performed using a pulmonary metastasis model. BALB/c-nu mice were grouped as follows and all mice were inoculated with 1 × 10^6^ cells/100 μl through the tail vein: HeLa-sh-NC (*n* = 5); and HeLa-sh-CENPK (*n* = 5). Four weeks after injection, the formation of lung metastasis was evaluated under light and fluorescent microscopy.

The group assignment was the same in the mice inoculated with SiHa cells except carboplatin (30 mg/kg, ip once a week for 4 weeks) [20] was substituted for cisplatin in the SiHa-sh-NC + carboplatin and SiHa-sh-CENPK + carboplatin groups. A total of 144 BALB/c-nu female mice (4–5-weeks-old) were used in this study.

### Luciferase reporter assay

The assays were carried out, as described in our previous study [21]. The Wnt signaling and p53 signaling activity assays were carried out using the luciferase assay system. Cervical cancer cells were co-transfected with TOPFlash or FOPFlash with pRL (Millipore Corporation, Billerica, MA, USA) to detect Wnt signaling. Cervical cancer cells were transfected with pGL4 luciferase reporter vector (Promega Corporation, Madison, WI, USA) harboring a p53 response element to detect p53 signaling. Cervical cancer cells were transfected with pmirGLO luciferase reporter vector (Promega Corporation) harboring 3’-UTR of *CENPK* to detect the impact of ZC3H13 on *CENPK* transcriptional levels. After transfection for 48 h, cells were collected and subjected to luciferase activity measurement using the Dual-Luciferase Reporter Assay System (Promega Corporation) on a BioTek luminometer.

### Co-immunoprecipitation (Co-IP)

Co-IP was performed with a Pierce Co-IP kit (Thermo Scientific, Shanghai, China), according to the manufacturer’s instructions. Briefly, total proteins were lysed from cervical cancer cells and the protein concentration was quantified. Proteins (5 mg) were then incubated with specific antibodies or IgG, which was used as a negative control. The enriched proteins were eluted and used for Western blotting.

### Chromatin immunoprecipitation (ChIP)

The ChIP assays were carried out with a ChIP assay kit (Thermo Scientific, Shanghai, China) according to the manufacturer’s instructions. Briefly, chromatin from cervical cancer cells was crosslinked, extracted, and clipped with *Micrococcal* Nuclease to generate DNA fragments. Immunoprecipitation was performed with specific antibodies (Additional file 1: Table S2) or IgG, which served as a negative control. The DNA fragments were eluted, purified, and subjected to PCR and/or qPCR.

### Cycloheximide chase assay

The cycloheximide chase assay was performed as described in our previous study [22]. Cervical cancer cells were subjected to treatment with cycloheximide (50 µg/ml) at the indicated time. Subsequently, proteins were collected and quantified using a BCA protein assay kit (Thermo Scientific, Waltham, MA, USA). The proteins were further subjected to Western blotting, and signals were recorded for evaluating the protein half-life.

### Cell fractionation assay

The cell fractionation assay was carried out with NE-PER™ Nuclear and Cytoplasmic Extraction Reagents (Thermo Scientific, Shanghai, China), as described in our previous study [22]. Cervical cancer cells were sequentially treated with ice-cold CER I and the CER II extraction reagent. The supernatant containing cytoplasmic extract was collected after centrifugation. The pellet was further incubated with NER extraction reagent. After centrifugation, the supernatant containing nuclear extract was retained. The collected proteins were further subjected to Western blotting.

### Methylated RNA immunoprecipitation (MeRIP)

According to the manufacturer’s instructions, MeRIP was performed using a MeRIP m^6^A kit (Millipore Corporation). Briefly, RNAs were extracted from cervical cancer cells and subjected to fragmentation followed by immunoprecipitation with magnetic beads conjugated with m^6^A antibody or IgG, which served as a negative control. The enriched RNAs were subjected to reverse transcription after RNA elution and purification. The synthesized cDNAs were further analyzed by qPCR and/or PCR.

### Patient tissues

One hundred-nineteen paraffin-embedded cervical cancer specimens and 35 paraffin-embedded adjacent normal tissues in tissue chips (HUteS154Su01) were purchased from Shanghai Outdo Biotech (Shanghai, China). All the patients underwent surgery and had a confirmed pathologic diagnosis. Clinical data were extracted from the patients’ medical records. Some clinicopathological characteristics, such as histological grade and Ki67 status, were not available for all patients. Ethics approval and patient written consent were obtained from the Ethics Committee of Shanghai Outdo Biotech Corporation (YB M-05–02). The procedures used in this study adhered to the tenets of the Declaration of Helsinki.

### Immunohistochemistry (IHC)

Paraffin-fixed Sects. (4 μm) from tissues were subjected to deparaffinization, rehydration, and antigen retrieval in citrate buffer. After eradicating endogenous peroxidase activity with 3% H_2_O_2_ and blocking non-specific antigens with goat serum, the sections were subjected to incubation with specific antibodies (Additional file 1: Table S2). The section signals were measured using DAB substrate (Maixin Biotech. Corporation, Fuzhou, China), and staining intensities were evaluated as previously described [23].

### Bioinformatics analysis

Bioinformatics analysis was carried out as described in our previous study [24]. The mRNA-Seq (HTSeq-FPKM) and genomic data from the cervical squamous cell carcinoma and endocervical adenocarcinoma (CESC) dataset in The Cancer Genome Atlas (TCGA) database, together with clinical data were used to perform differential expression analysis with the limma R package, and correlation analysis, survival analysis, gene set enrichment analysis (GSEA), gene set variation analysis (GSVA), and unsupervised PAM clustering analyses with the ConsensusClusterPlus R package [25]. The Maftools R package was used for defining mutation co-occurrence/mutually exclusive. The platinum drug resistance- and the radioresistance-associated gene sets were established based on KEGG websites and a previous study [26], respectively. Samples were classified into high and low expression groups according to the best cut-off value for survival analysis in TCGA database.

### Statistical analysis

The continuous variables are calculated as the mean ± SD from at least three independent experiments and categorical variables are expressed as *n* (%). The data were analyzed using SPSS 22.0 or RStudio. A parametric generalized linear model with random effects for the growth curve, the Student’s two-tailed *t*-test, and the Wilcoxon rank-sum tests for two groups, and one-way ANOVA with LSD-*t* test for multiple groups were used for detecting statistical significance. The correlation analyses were performed using the chi-square and Spearman’s rank correlation tests. Kaplan–Meier survival curves were plotted and log-rank tests were applied for exploring a survival difference. Cox regression models were adopted to identify the relationship between CENPK and the survival time of cervical cancer patients. All statistical tests were two-sided and a *P* value < 0.05 was considered statistically significant.

## Results

### Bioinformatics analyses revealed the involvement of m^6^A modification in cervical cancer progression

To better understand whether and how m^6^A regulators contribute to cervical cancer progression, we first identified 9 m^6^A writers (*WTAP*, *ZC3H13*, *METTL3*, *METTL14*, *METTL16*, *VIRMA*, *RBM15B*, *RBM15*, and *CBLL1*), 15 m^6^A readers (*FMR1*, *hnRNPA2B1*, *hnRNPC*, *YTHDF1/2/3*, *YTHDC1/2*, *LRPPRC*, *IGF2BP1*, *IGF2BP2*, *IGF2BP3*, *RBMX*, *EIF3A*, and *ELAVL1*), and 2 m^6^A erasers (*FTO* and *ALKBH5*) that were previously reported to have pathogenic roles in other human cancer types [7, 8]. Using the TCGA CESC database, we identified copy number variations (CNVs) and somatic mutations in the m^6^A-related genes because amplification or deletions that affect copy number can also affect expression of the corresponding gene. This analysis showed that 21 m^6^A regulators harbored CNVs in cervical cancer (Additional file 2: Fig. S1). Among these genes, 13 of the 21 carried mutations at a frequency of 1–4%, while the remaining 8 genes had mutations at a frequency of < 1% (Additional file 2: Fig. S2a). Analysis of mutation co-occurrence indicated that several of these genes also carried mutations that significantly co-occurred with mutations in other m^6^A regulators (Additional file 2: Fig. S2b). RNA-seq data in the TCGA database further showed that these m^6^A regulators were differentially expressed in cervical cancer samples compared with the expression in adjacent normal tissues (Additional file 2: Fig. S2c), which suggested that CNVs and other somatic mutations could lead to dysregulation of the m^6^A-related genes in cervical cancer.

Continuing our analysis of possible relationships between m^6^A-related genes and cervical cancer in public datasets, we next focused on the potential clinical significance of these genes. Survival analyses indicated that 15 CNV-carrying genes had altered levels of expression that were significantly correlated with cervical cancer patient prognosis (Additional file 2: Fig. S3), whereas 6 of the genes showed no significant value as candidate prognostic indicators. Subsequent gene interaction network analysis indicated that 3 genes (*ZC3H13*, *METTL14*, and *CBLL1)* appeared to serve as network hubs, suggesting that dysregulation of m^6^A modification by these genes provided major contributions to the development and/or progression of cervical cancer (Additional file 2: Fig. S4a). Correlation analyses also revealed a strong association among these 21 m^6^A regulators (Additional file 2: Fig. S4b). Notably, unsupervised clustering of the 21 m^6^A-related gene expression patterns in different patients identified three distinct patterns of m^6^A modification (i.e., clusters A-C) associated with cervical cancer (Additional file 2: Fig. S4c, d). Survival analyses showed that the prognosis of patients with cluster B pattern generally had a better prognosis than patients exhibiting cluster A or C patterns (Additional file 2: Fig. S4e).

To determine the biological significance of these m^6^A modification patterns, we conducted GSVA to elucidate differentially-enriched KEGG pathways and GO terms in these clusters that are related to the m^6^A modification patterns identified in cervical cancer samples. The results showed that clusters A and C were enriched for canonical cancer signaling pathways and processes, such as the VEGF, mTOR, ERBB, MAPK, Wnt, TGF-β, hedgehog, and Notch pathways, as well as tight junctions, adherens junctions, cell cycle, non-homologous end joining, mitotic sister chromatid cohesion, mRNA processing, and related GO terms compared with samples exhibiting cluster B patterns (Additional file 2: Fig. S5), further indicating the involvement of m^6^A modification in cervical cancer progression.

### ***CENPK*** expression is correlated with aberrant m^6^A modification and tumorigenic gene expression in cervical cancer

We compared the differential expression data from these 21 genes with the m^6^A methylation data available in the m^6^A-Atlas database and the data obtained in our previous study of cervical cancer patients [23] to identify specific genes that could be dysregulated by m^6^A modification in cervical cancer. First, we performed differential expression analyses between m^6^A cluster B and C patterns, which identified 3628 differentially-expressed genes in different m^6^A clusters [fold-change ≥ 2.0 (geneset A); *P* < 0.05]. Furthermore, experimental evidence in the m^6^A-Atlas database (http://180.208.58.66/m6A-Atlas/) suggested the participation of 7617 genes with an m^6^A modification in HeLa cells (geneset B). Our previous study [23] also showed a possible correlation between 151 differentially-expressed genes and cervical cancer cell progression [fold-change ≥ 2.0 (geneset C); *P* < 0.05]. A total of 40 overlapping genes were obtained from the intersection of the 3 genesets (Additional file 2: Fig. S6a).

Subsequent GSVA revealed that pathways involving *CENPK* had the greatest overlap with m^6^A-modified clusters associated with cervical cancer. Specifically, *CENPK* was enriched for genes in pathways, such as Wnt signaling, DNA damage repair signaling, the cell cycle, and DNA replication (Additional file 2: Fig. S6b). *CENPK* expression was elevated in pan-cancer datasets compared to that in corresponding normal tissues based on TCGA (Additional file 2: Fig. S6c). Interestingly, GSEA showed that *CENPK* also participated in the modulation of platinum drug resistance more prominently than radioresistance (Additional file 2: Fig. S6d). In addition, correlation analyses showed a positive association between *CENPK* and stemness markers, including *EPCAM*, *CD133*, *SOX2*, and *OCT4* (Additional file 2: Fig. S6e). Taken together, these results indicated a strong correlation between *CENPK* expression and the dysregulation of m^6^A modifications in cervical cancer.

### ZC3H13-mediated m^6^A methylation upregulated ***CENPK*** mRNA to activate pro-tumorigenic functions

**Fig. 1:**
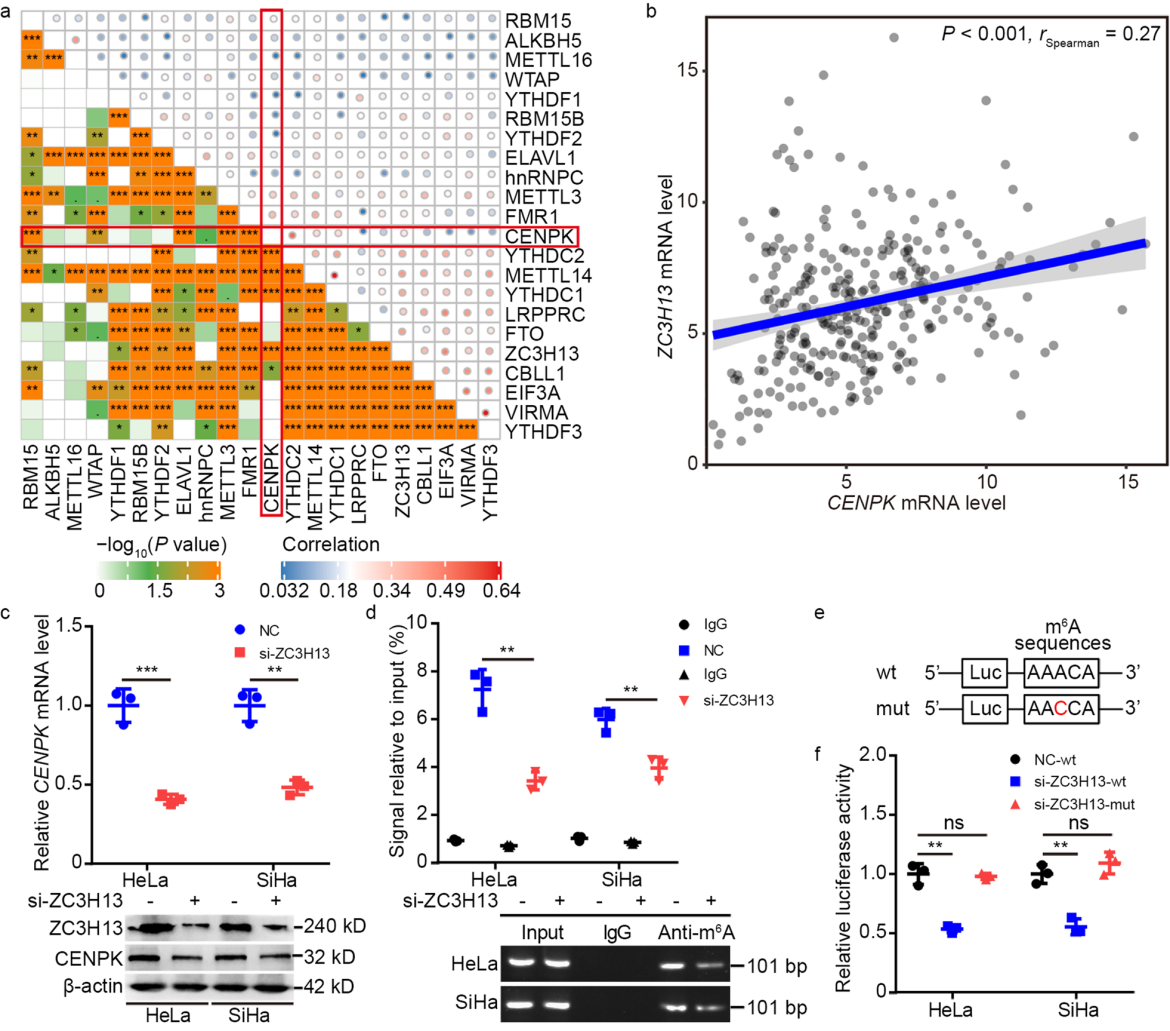
ZC3H13 participates in regulating m^6^A modification of *CENPK* mRNA. **a** Correlogram displaying the link between *CENPK* and 21 m^6^A regulators expression as per the TCGA CESC dataset. The red frame emphasizes the correlation between *CENPK* and 21 m^6^A regulators expression. **b** Relationship between *CENPK* and *ZC3H13* expression as per the TCGA CESC dataset. **c** qPCR and Western blotting determining the effect of *ZC3H13* knockdown on *CENPK* expression. **d** MeRIP assays were adopted to measure the m^6^A modification of *CENPK* mRNA. The enriched RNAs were subjected to reverse transcription and further analyzed by qPCR and PCR. **e** Bioinformatics analysis predicting m^6^A sequences within the *CENPK* 3′-UTR. **f** Luciferase reporter assays were carried out for validating the effect of *ZC3H13* knockdown on the post-transcriptional repression of *CENPK* in HeLa and SiHa cells. Data are represented as the mean ± SD. ^**^P < 0.01; ^***^P < 0.001; ns non-significant

We further examined whether CENPK expression was regulated by m^6^A RNA methylation based on the results of our bioinformatics analyses. The above mentioned survival analysis showed 9 m^6^A regulators (*EIF3A*, *hnRNPC*, *LRPPRC*, *RBM15B*, *VIRMA*, *WTAP*, *YTHDF2*, *YTHDF3*, and *ZC3H13*) with the potential to confer poor patient survival (Additional file 2: Fig. S3). Indeed, *CENPK* expression was correlated with *CBLL1*, *ELAVL1*, *FMR1*, *METTL3*, *METTL14*, *RBM15*, *WTAP*, *YTHDC1*, *YTHDC2*, and *ZC3H13* expression (Fig. 1a, b). Thus, *ZC3H13* and *WTAP* were correlated with poor patient survival and *CENPK* expression, and of these two candidates, *CENPK* had a more prominent correlation with *ZC3H13* expression than with *WTAP* expression. Because CENPK has a potential oncogenic role in cervical cancer, we speculated that ZC3H13 might be responsible for m^6^A methylation of *CENPK* mRNA. To test this possibility, we generated HeLa and SiHa cells with *ZC3H13* knockdown by siRNA (si-ZC3H13), which downregulated *CENPK* expression (Fig. 1c). In addition, MeRIP assays confirmed that *CENPK* mRNA was enriched by anti-m^6^A antibody in HeLa and SiHa cells, while m^6^A modification of *CENPK* mRNA was reduced in si-ZC3H13 cells (Fig. 1d). Moreover, DART-seq data in the RMVar database (http://rmvar.renlab.org) identified a site in the 3’ UTR of *CENPK* mRNA on chr5:65518395(-) as an m^6^A modification site (Fig. 1e). Luciferase reporter assays suggested that even though *ZC3H13* suppression impaired the transcription of wild-type *CENPK*, non-synonymous cytosine-to-adenosine conversion mutations at this m^6^A site abolished the downregulation (Fig. 1f). These results thus indicated that CENPK expression was modulated by ZC3H13-associated m^6^A modification.

Based on these findings, we further investigated the effects of ZC3H13 on CENPK-mediated downstream signaling. Considering its overlap with cervical cancer-enriched pathways (vide supra), we focused on Wnt and p53 signaling. TOP/FOP luciferase reporter and dual-luciferase assays revealed that *ZC3H13* knockdown decreased Wnt signaling activity and elevated p53 signaling activity in HeLa and SiHa cells, but this effect was abolished by overexpressing *CENPK* (ov-CENPK) (Additional file 2: Fig. S7a, b). Indeed, tumorsphere and colony formation, and Transwell, EdU, and immunofluorescence staining assays showed that *ZC3H13* knockdown had a suppressive effect on cervical cancer stemness, chemoresistance, metastasis, and cell proliferation that was reversed by ov-CENPK in HeLa and SiHa cells (Additional file 2: Fig. S7c-i). These results collectively demonstrated that ZC3H13 functioned as a regulator of CENPK expression through m^6^A RNA methylation and that these genes functioned together in facilitating cervical cancer progression.

### CENPK expression was correlated with cervical cancer pathology

**Fig. 2:**
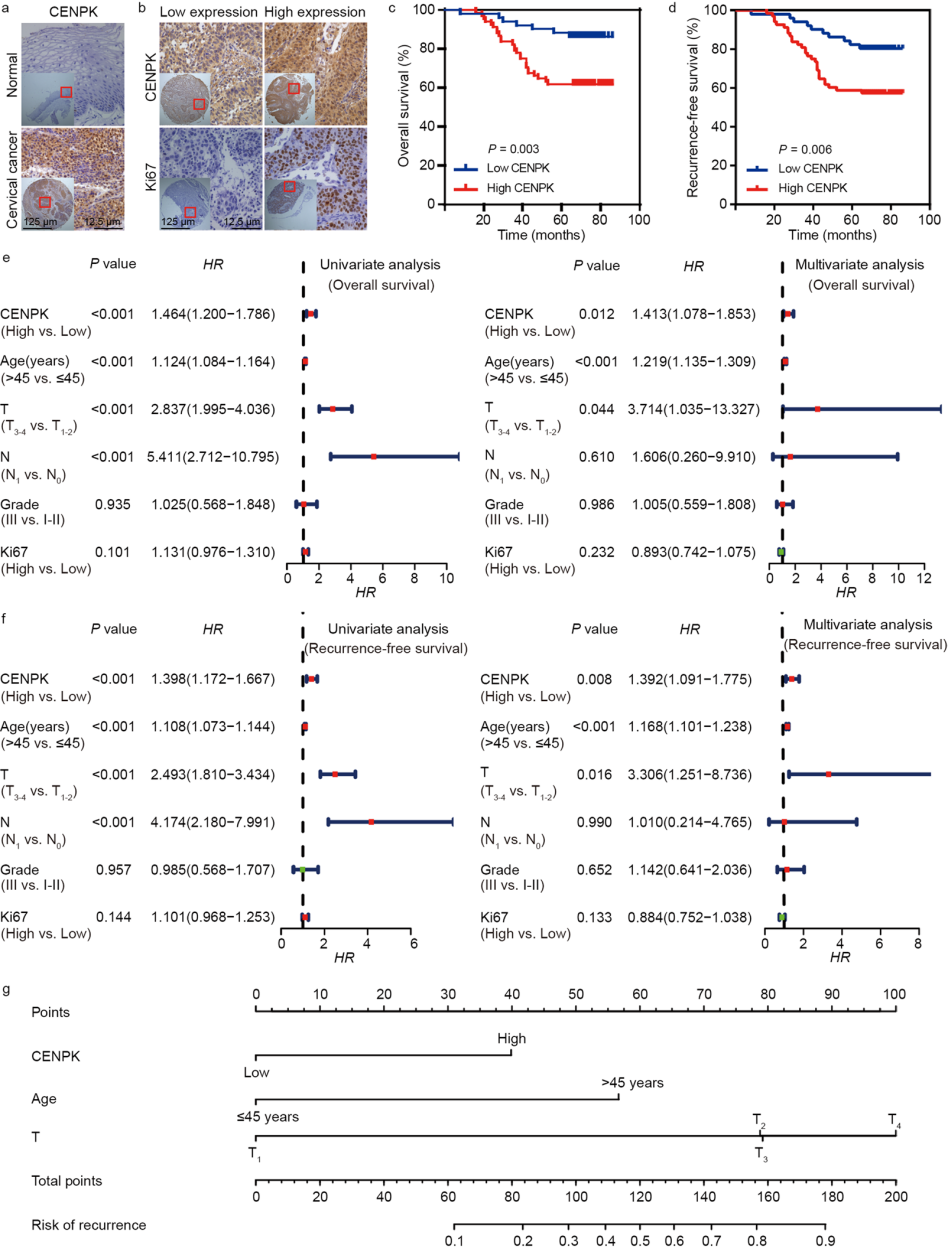
CENPK expression is elevated in cervical cancer and confers poor patient prognosis. **a** Representative images showing the differential CENPK expression between cervical cancer and adjacent normal tissues. **b** Representative images showing the relationship between CENPK and Ki67 expression in cervical cancer. **c** Kaplan–Meier survival analysis disclosing the overall survival of cervical cancer patients according to CENPK expression. **d** Kaplan–Meier survival analysis displaying the recurrence-free survival of cervical cancer patients according to CENPK expression. **e** Univariate and multivariate analyses were adopted for correlating CENPK expression, clinicopathological characteristics, and overall survival of cervical cancer patients. **f** Univariate and multivariate analyses were performed to correlate CENPK expression, clinicopathological characteristics, and recurrence-free survival of cervical cancer patients. **g** Development of a nomogram for predicting cancer recurrence of cervical cancer patients. HR hazard ratio

To further establish the central role of CENPK in cervical cancer, we conducted IHC analysis of 119 cervical cancer samples and 35 adjacent normal tissues. The results showed elevation of CENPK expression in cervical cancer compared with adjacent normal tissues (Fig. 2a). Moreover, IHC staining of CENPK protein indicated a positive association with Ki67 protein levels (Fig. 2b) and was positively correlated with cancer recurrence (Additional file 1: Table S3).

Survival analysis suggested that cervical cancer patients with high CENPK expression exhibited poor overall survival (Fig. 2c). Because CENPK expression was correlated with cancer recurrence, we further investigated the relationship between CENPK expression and recurrence-free survival in cervical cancer patients. Survival analysis of cervical cancer patients confirmed that high CENPK expression was a predictor of poor recurrence-free survival, which is consistent with correlation analysis (Fig. 2d). Quantitative analysis of IHC staining, followed by univariate and multivariate COX hazard analyses, indicated that CENPK expression was an independent and unfavorable prognostic indicator for overall and recurrence-free survival in cervical cancer patients (Fig. 2e, f). A nomogram showing the role of CENPK, age, and T classification in predicting cancer recurrence in cervical cancer patients is shown in Fig. 2g. When stratified by age or T classification, CENPK expression remained correlated with overall and recurrence-free survival (Additional file 2: Fig. S8a, b). Consistent with these results, correlation analyses suggested a positive association between *β-catenin*, *Ki67*, and *CENPK* expression (Additional file 2: Fig. S8c). Moreover, *ZC3H13* expression was positively associated with *EPCAM*, *CD133*, *β-catenin*, *Ki67*, *N-cadherin*, and *CCND1* expression in the TCGA database (Additional file 2: Fig. S8d). Taken together, these data showed a clear role for CENPK in cervical cancer development and indicated the clinical value as a potential biomarker.

### Silencing *CENPK* suppressed cervical cancer stemness, chemoresistance, metastasis, and proliferation

**Fig. 3:**
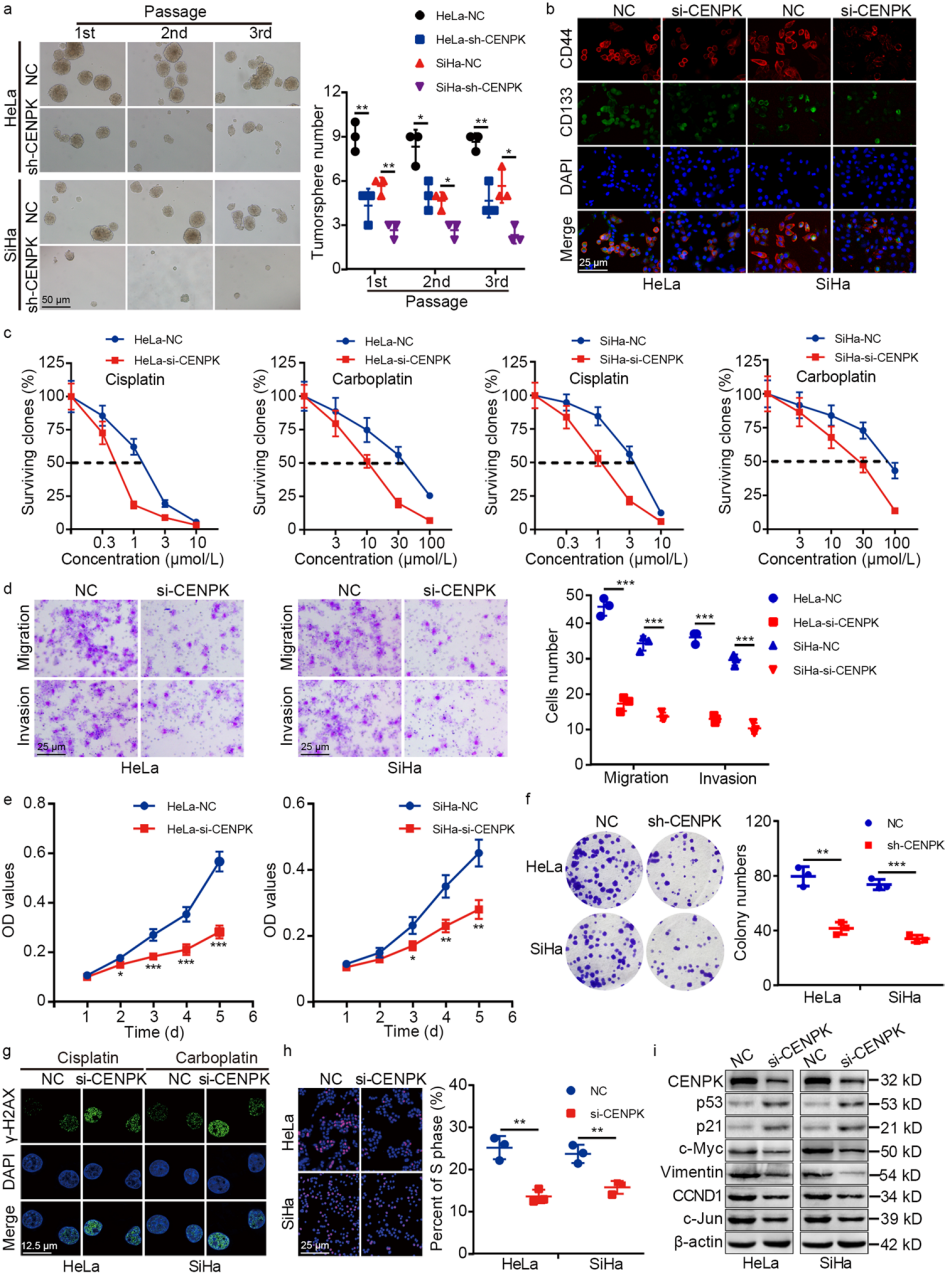
CENPK promotes cervical cancer stemness, chemoresistance, metastasis, and proliferation. Tumorsphere formation (**a**) and immunofluorescence assays (**b**) were adopted to measure stemness of *CENPK*-depleted HeLa and SiHa cells and the control cells. **c** Clonogenic assays were adopted to estimate chemoresistance of *CENPK*-depleted HeLa and SiHa cells and the control cells treated with cisplatin and carboplatin. **d** Transwell assays were performed to elucidate the migration and invasion of *CENPK*-silenced HeLa and SiHa cells and the control cells. MTT assays (**e**), and colony-formation assays (**f**) were applied to evaluate the proliferation of *CENPK*-silenced HeLa and SiHa cells and the control cells. **g** Immunofluorescence was adopted for detecting the expression of γ-H2AX (Ser139) in *CENPK*-silenced HeLa treated with cisplatin (10 μmol/L for 24 h), *CENPK*-silenced SiHa cells treated with carboplatin (100 μmol/L for 24 h), and control cells. **h** EdU incorporation assays were used for elucidating DNA replication of *CENPK*-depleted HeLa and SiHa cells and the control cells. **i** Western blotting analysis of the expression of proteins associated with stemness (c-Myc), DNA damage repair (p53), epithelial-mesenchymal transition (Vimentin), and DNA replication (p21, CCND1 and c-Jun) in *CENPK*-depleted HeLa and SiHa cells and control cells. Data are represented as the mean ± SD. ^*^P < 0.05, ^**^P < 0.01, ^***^P < 0.001

Given this validation of CENPK contribution to cervical cancer, we sought to determine how CENPK affected cell stemness, chemoresistance, metastasis, and proliferation. To this end, *CENPK* was silenced by shRNA (sh-CENPK) or siRNA (si-CENPK) in HeLa and SiHa cell lines. Tumorsphere formation assays and immunofluorescence staining of cervical cancer stem cell markers (CD133 and CD44) indicated that *CENPK* knockdown reduced stemness of HeLa and SiHa cells (Fig. 3a, b). Clonogenic assays revealed that *CENPK* suppression impaired cisplatin and carboplatin resistance (Fig. 3c), Transwell assays showed impaired migration and invasion (Fig. 3d), and MTT and colony formation assays revealed inhibited cell growth by sh-CENPK or si-CENPK in HeLa and SiHa cells (Fig. 3e, f). Moreover, immunofluorescence staining of γ-H2AX (Ser139) showed that downregulation of *CENPK* led to enhanced DNA damage in cervical cancer cells treated with platinum-based drugs (Fig. 3g). Consistent with the bioinformatics results, EdU assays suggested that *CENPK* knockdown decreased the percentage of cells in the S phase and inhibited DNA replication in HeLa and SiHa cells (Fig. 3h). In addition, the expression of DNA damage repair-associated proteins (p53 and p21) was enhanced, while cell stemness, epithelial-mesenchymal transition (EMT), and DNA replication-associated proteins (c-Myc, Vimentin, CCND1, and c-Jun) were suppressed in si-CENPK cells (Fig. 3i; Additional file 2: Fig. S9). These results indicated that high CENPK expression stimulated cervical cancer stemness, chemoresistance, metastasis, and proliferation.

**Fig. 4:**
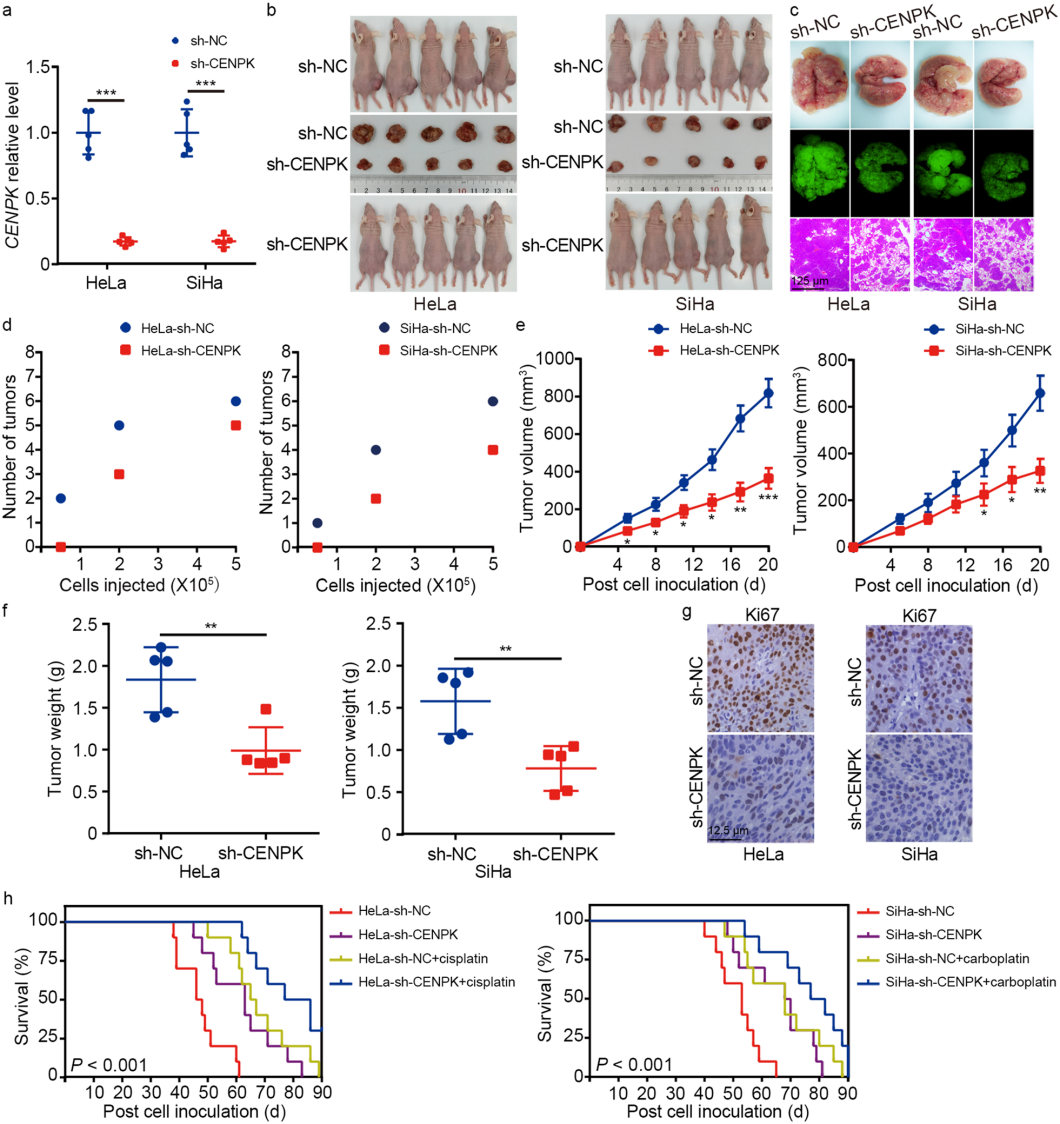
CENPK enhances tumorigenic functions of cervical cancer cells in vivo. **a** qPCR analyses of *CENPK* mRNA levels in HeLa and SiHa-derived xenografts with *CENPK* silencing and controls. **b-d** Impact of CENPK on cervical cancer proliferation (*n* = 5/group), metastasis (*n* = 5/group), and tumor formation ability (*n* = 6/group) was detected by establishing the xenograft mouse models. Tumor volume (**e**) and tumor weight (**f**) were calculated and recorded. **g** Xenograft tumors were subjected to detection of Ki67 expression by immunohistochemistry (IHC). **h** Survival analyses were performed to compare the overall survival time of the mice in the *CENPK*-silenced group (*n* = 10), the cisplatin/carboplatin-treated group (*n* = 10), the *CENPK* knockdown plus cisplatin/carboplatin-treated group (*n* = 10), and the controls (*n* = 10). Data are represented as the mean ± SD. ^**^P < 0.01, ^***^P < 0.001

We established cervical cancer models in BALB/c-nu mice harboring wild-type or stably-silenced *CENPK* to determine how CENPK affected cancer progression in vivo. We confirmed that *CENPK* expression was decreased in tumors treated with *CENPK*-targeted shRNA (Fig. 4a). Mice with downregulated CENPK in HeLa and SiHa cells exhibited decreased tumor formation, fewer lung metastases, and slower growth rates than the corresponding controls (Fig. 4b-f). IHC staining also showed lower Ki67 expression in sh-CENPK tumors compared to scramble shRNA control tumors (Fig. 4g). Moreover, *CENPK* silencing prolonged the overall survival time of model mice, and *CENPK* suppression led to synergistic effects with chemotherapy to further extend the mouse survival time compared with that of mice receiving chemotherapy only (Fig. 4h).

### CENPK activated Wnt signaling and inactivated p53 signaling via SOX6 in cervical cancer

**Fig. 5:**
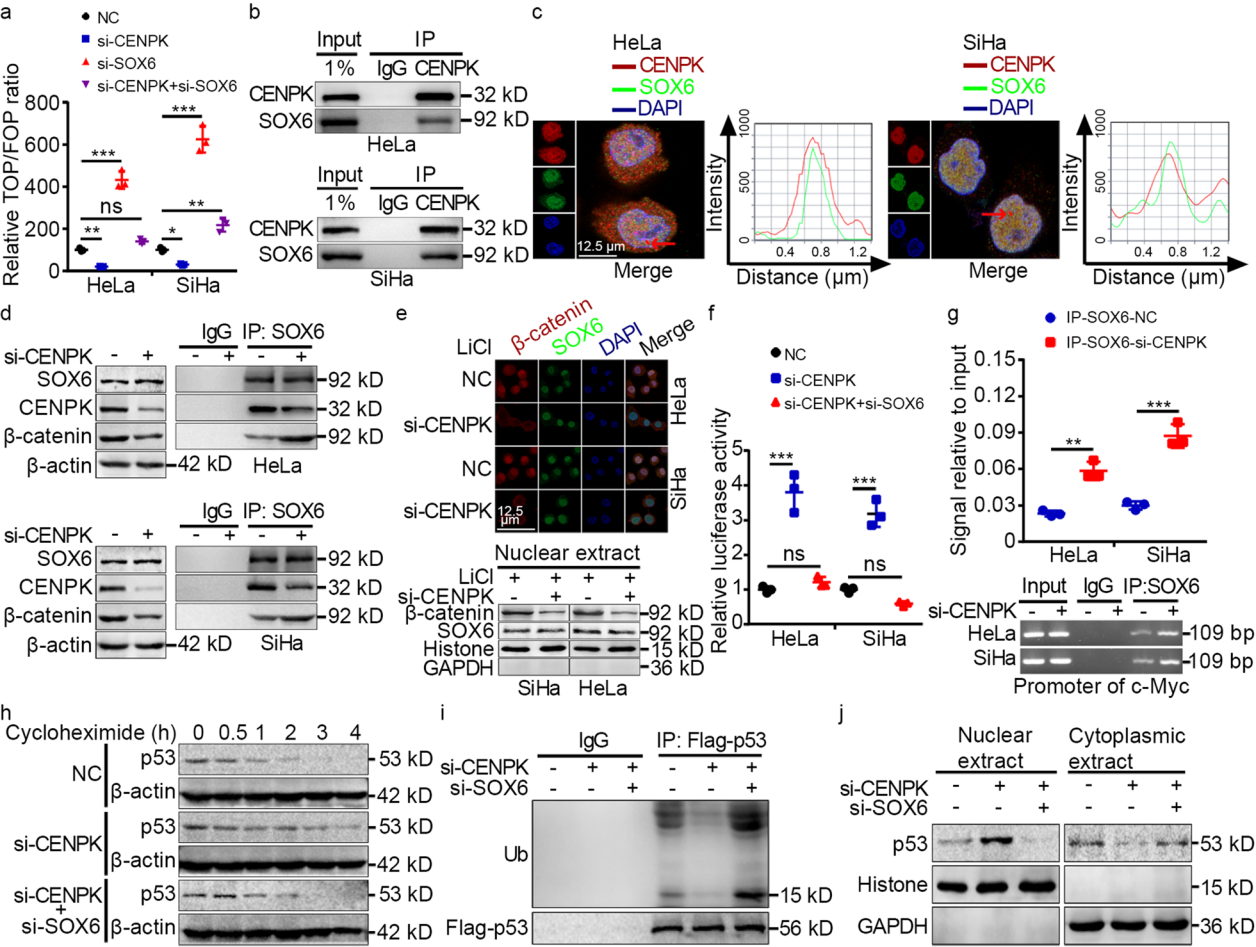
CENPK interacts with SOX6 to activate Wnt signaling and inactivate p53 signaling in cervical cancer. **a.** TOP/FOP luciferase reporter assays were conducted for measuring the impact of CENPK and SOX6 on Wnt signaling activity. **b.** Co-immunoprecipitation analyses displaying the interaction between CENPK and SOX6. **c.** Immunofluorescence co-staining showing the colocalization of CENPK and SOX6. The red bars indicated by the arrows represent the colocalization of CENPK and SOX6. **d.** Co-immunoprecipitation analyses displaying the effect of CENPK knockdown on the interaction between CENPK and SOX6, and the impact of CENPK knockdown on the interplay between SOX6 and β-catenin. **e.** Immunofluorescence co-staining and cell fractionation assays showing the effect of CENPK on the expression and nuclear translocation of β-catenin and SOX6 in HeLa and SiHa cells incubated with lithium (30 nmol/L). **f.** Luciferase reporter assays were performed for elucidating the effect of CENPK and SOX6 on p53 signaling activity. **g.** Chromatin immunoprecipitation analyses were applied to estimate the impact of CENPK on SOX6-mediated *c-Myc* transcription. **h.** Cycloheximide chase assays assessing the impact of CENPK and SOX6 on p53 stability in HeLa cells. **i.** Co-immunoprecipitation analyses verifying the impact of CENPK and SOX6 on p53 ubiquitination in HeLa cells treated with MG132. **j.** Cell fractionation assays validating the impact of CENPK and SOX6 on p53 nuclear export in HeLa cells. Data are represented as the mean ± SD. ^*^P < 0.05, ^**^P < 0.01, ^***^P < 0.001; ns non-significant; IP immunoprecipitation; LiCl lithium

We next determined the specific mechanisms by which CENPK exerted oncogenic effects. A review of the BioGRID database (https://downloads.thebiogrid.org/BioGRID) suggested that SOX6 potentially interacted with CENPK, and our above bioinformatics analysis indicated that CENPK modulated Wnt signaling (Additional file 2: Fig. S6). Together with TOP/FOP-flash assays, which confirmed CENPK promoted Wnt signaling (Fig. 5a), and previous studies that showed SOX6 activated p53 signaling and inactivated Wnt signaling [27] while also suppressing cervical cancer progression [28], we selected SOX6 as a candidate protein interaction partner of CENPK. Co-IP assays verified that CENPK interacted with SOX6 (Fig. 5b), and immunofluorescent staining confirmed the co-localization of CENPK and SOX6 in HeLa and SiHa cells (Fig. 5c).

SOX6 is known to inhibit the transcription of target genes regulated by β-catenin via direct interaction with β-catenin to inactivate Wnt signaling [27]. Moreover, TOP/FOP-flash assays suggested that *SOX6* knockdown had regulatory functions in CENPK-mediated Wnt signaling (Fig. 5a). These results thus implied that CENPK-SOX6 interaction might affect interactions between SOX6 and β-catenin. Co-IP analyses subsequently revealed an interaction between SOX6 and β-catenin, and this SOX6 interaction with β-catenin was enhanced in si-CENPK cells with decreased CENPK-SOX6 binding (Fig. 5d). Moreover, immunofluorescence staining and cell fractionation assays suggested that *CENPK* knockdown inhibited β-catenin expression and nuclear translocation; however, no impact on SOX6 localization or expression by CENPK was detected (Fig. 5e).

We next investigated the effects of CENPK on p53 signaling in light of the report that SOX6 activates p53 signaling by increasing protein stability via transcriptional suppression of *c-Myc* in HeLa cells [28]. Dual-luciferase assays showed increased p53 signaling in *CENPK* knockdown cells, whereas *SOX6* silencing abolished this effect (Fig. 5f). ChIP assays indicated that SOX6 binding to the *c-Myc* transcription regulatory region was increased in HeLa and SiHa cells (Fig. 5g). Furthermore, cycloheximide chase assays, Co-IP analyses, and cell fractionation assays all confirmed that *CENPK* knockdown led to increased p53 stability and inhibition of p53 ubiquitination and nuclear export, all of which were reversed by *SOX6* knockdown (Fig. 5h-j). These data suggested that SOX6 mediated the effects of CENPK on Wnt and p53 signaling.

### CENPK promoted tumorigenic functions of cervical cancer cells via Wnt and p53 signaling

We then investigated how CENPK function in cervical cancer was mediated by Wnt and p53 signaling. Immunofluorescence staining, tumorsphere formation, clonogenic, MTT, and EdU assays collectively showed that *β-catenin* overexpression or *p53* knockdown reversed the inhibitory effects of *CENPK* knockdown on cell stemness, chemoresistance, migration, invasion, and proliferation in HeLa and SiHa cells (Additional file 2: Fig. S10a-f). Immunofluorescence staining and Western blotting further showed that *β-catenin* overexpression or *p53* knockdown abolished the diverse effects of *CENPK* knockdown on DNA damage repair, cell stemness, EMT, and DNA replication-associated gene expression [i.e., γ-H2AX (Ser139), p53, p21, c-Myc, Vimentin, CCND1, and c-Jun] (Additional file 2: Fig. S10g, h). These results thus showed that the pro-tumorigenic effects of CENPK were mediated by Wnt and p53 downstream regulatory targets.

## Discussion

Stemness, chemoresistance, metastasis, and tumor size are known to contribute to cancer recurrence and poor prognosis in cervical cancer patients [29, 30]. Notably, m^6^A RNA methylation has been recently shown to play an important role in controlling biological processes required for the development and progression of human cancers, such as cell stemness, drug resistance, metastasis, and proliferation [31]. In the current study, we identified clear links between m^6^A RNA methylation and cervical cancer using bioinformatics analyses, thus suggesting that specific m^6^A modification patterns play a critical role in cervical cancer. Specifically, our work showed that m^6^A methylation regulated *CENPK* expression, and subsequently enhanced Wnt signaling and attenuated p53 signaling to modulate the expression of key regulators of DNA damage repair, EMT, and DNA replication. These downstream effects thus promoted the tumorigenic characteristics of cervical cancer cells, such as stemness, chemoresistance, metastasis, and proliferation. In contrast, *CENPK* downregulation resulted in delayed progression of cervical cancer in vitro and in vivo. Moreover, high CENPK expression was positively correlated with poor overall and recurrence-free survival in cervical cancer patients.

Distinct m^6^A RNA methylation patterns have been shown to serve as determining factors in cancer immunity and patient prognosis [25]. Our bioinformatics analyses identified three m^6^A RNA methylation patterns associated with aberrant activity due to CNVs in 21 different m^6^A regulators. Because CNV affected the expression of these 21 m^6^A-related genes, it was reasonable to hypothesize that expression of the 21 m^6^A-related genes significantly contributed to cancer progression, as shown in our previous work [24]. The abnormal expression of these m^6^A-related genes and their prognostic value in cervical cancer further support the regulatory role of m^6^A RNA methylation in driving cancer development [25]. Based on the association between dysregulation of m^6^A methylation and cervical cancer, we were able to identify *CENPK* as a primary target of m^6^A RNA methylation that was also correlated with cancer development.

Previous studies have demonstrated that ZC3H13 provided regulatory effects on stem cell self-renewal and oncogenesis of cervical cancer [32, 33]. Our findings further illustrated how ZC3H13 functions in promoting the tumorigenic properties of cervical cancer cells via CENPK. Consistent with findings from a previous study that ablation of ZC3H13 caused a global reduction of m^6^A modification, especially at the 3’ ends of mRNA [34], we found that CENPK expression was modulated by ZC3H13-associated m^6^A modification in the 3’-UTR of *CENPK* mRNA in the current study. Moreover, we confirmed that ZC3H13 enhanced CENPK expression, supporting the bioinformatic findings of m^6^A-mediated dysregulation of *CENPK* and the correlation between *ZC3H13* and *CENPK* in cervical cancer. Our results subsequently verified that ZC3H13 was responsible for CENPK disturbance of Wnt and p53 signaling, suggesting that these genes function in concert to drive cervical cancer progression. Considering the global effects of ZC3H13 on m^6^A modification, we noticed that the proposed Wnt and p53 signaling may not be the only axis responsible for ZC3H13-mediated cervical cancer progression.

In contrast, SOX6 is known to function in tumor suppression of several cancers [35–37], including cervical cancer [38]. Moreover, SOX6 has been shown to inhibit Wnt/β-catenin signaling, but activate p53 signaling [39, 40]. Here, we demonstrated that CENPK interacted with SOX6 to impair interactions between SOX6 and β-catenin, as well as SOX6-mediated suppression of *c-Myc* transcription, which resulted in activating the Wnt signaling pathway and inactivating the p53 pathway. In addition, the positive regulation of β-catenin expression by CENPK and its subsequent nuclear translocation contributed to Wnt pathway activation, which is consistent with findings reported in previous studies [21, 41]. Although p53 has been shown to positively regulate cancer cell resistance to platinum [42], Wnt signaling could also potentially mediate cancer cell resistance to platinum through several mechanisms, such as regulation of cancer stemness, EMT, and DNA damage repair [16, 43]. In addition, both the Wnt and p53 pathways have been shown to participate in regulating cancer cell stemness, metastasis, and proliferation [22, 44]. In our investigation, we further linked the dysregulation of these pathways with cervical cancer progression through the effects of CENPK in enhancing stemness, DNA damage repair (i.e., cisplatin and carboplatin resistance), EMT (necessary for tumor metastasis), and DNA replication processes related to cell proliferation. It should be noted, however, that our investigation using clinical samples was a single-center study, and the side effect of *CENPK* knockdown on the normal tissues was undetermined. Conducting a large-scale and multi-center study and constructing *CENPK* knockout mice to explore the possible impact caused by *CENPK* loss might overcome the limitations of our work.

## Conclusions

**Fig. 6:**
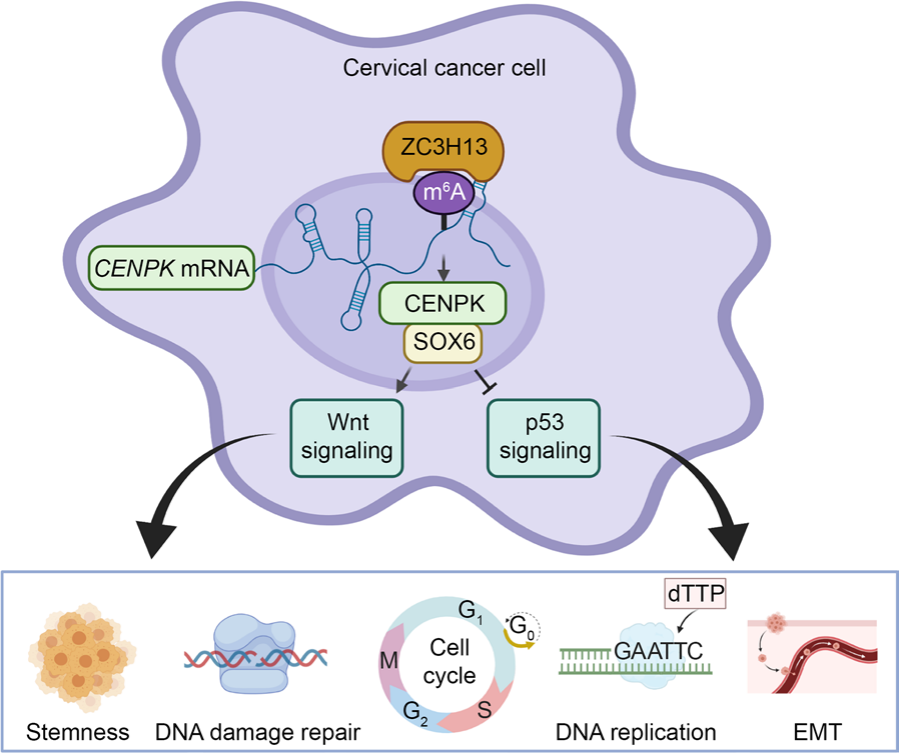
Working model of the ZC3H13-CENPK-SOX6 regulatory axis in controlling stemness, DNA damage repair, cell cycle, DNA replication, and EMT through Wnt and p53 signaling

Overall, the present study revealed a novel ZC3H13-CENPK-SOX6-p53/Wnt regulatory axis in cervical cancer development and progression through activation of tumorigenic functions, leading from m^6^A regulation of RNA. Furthermore, this work identified a previously unrecognized mechanism by which CENPK interfered with the interaction between SOX6 and β-catenin by direct binding with SOX6. This disruption of SOX6 activity resulted in increased β-catenin expression and nuclear translocation, and enhanced p53 ubiquitination and nuclear export, thereby upregulating Wnt signaling and downregulating p53 signaling. Finally, our results indicated that the m^6^A regulator (ZC3H13) was responsible for stimulating CENPK/SOX6 activation of Wnt/β-catenin and suppression of the p53 axis in this regulatory axis for cervical cancer progression (Fig. 6). This work thus demonstrated a role for m^6^A RNA methylation in controlling specific signaling pathways and highlighted a theoretic framework for clinical application of CENPK as a prognostic indicator and as a novel target for cervical cancer treatments.

## Supplementary Information

**Additional file 1.** **Table S1** Sequences used in this study. **Table S2** A list of antibodies used for ChIP, Co-IP, IF, IHC, and WB. **Table S3** Correlation between CENPK expression and the clinicopathological characteristics of cervical cancer patients.

**Additional file 2.** **Fig. S1** DNA copy number variation of 21 m^6^A regulators in cervical cancer based on TCGA database. **Fig. S2** Genetic and expression variation profiles of m^6^A regulators in cervical cancer based on TCGA database. **Fig. S3** Survival analysis of cervical patients in TCGA database. **Fig. S4** Unsupervised clustering of m^6^A regulators as per TCGA CESC dataset. **Fig. S5** Gene set variation analysis identifies the involvement of biological characteristics in distinct m^6^A methylation patterns. **Fig. S6** Bioinformatics analyses of m^6^A-modified *CENPK* based on the microarray and TCGA data. **Fig. S7** ZC3H13 augments pro-tumorigenic functions of cervical cancer cells through CENPK-modulated Wnt and p53 signaling. **Fig. S8** CENPK confers poor patient prognosis by stratified analysis and its relationship with related genes expression. **Fig. S9** CENPK promotes cervical cancer stemness, chemoresistance, metastasis, and proliferation. **Fig. S10** CENPK augments pro-tumorigenic functions of cervical cancer cells through Wnt and p53 signaling.

## Abbreviations

CENPK: Centromere protein K
CESC: Cervical squamous cell carcinoma and endocervical adenocarcinoma
ChIP: Chromatin immunoprecipitation
CI: Confidence interval
CNVs: Copy number variations
Co-IP: Co-immunoprecipitation
DMEM: Dulbecco’s modified Eagle medium
EMT: Epithelial-mesenchymal transition
GSEA: Gene set enrichment analysis
GSVA: Gene set variation analysis
*HR*: Hazard ratio
IHC: Immunohistochemistry
MeRIP: Methylated RNA Immunoprecipitation
m^6^A: *N*^*6*^-Methyladenine
qPCR: Quantitative real-time PCR
RT-PCR: Reverse transcription-polymerase chain reaction
TCGA: The Cancer Genome Atlas

## Declarations

### Availability of data and materials

The datasets used and/or analyzed during the current study are available from the corresponding author on reasonable request.
