# Supplementary material for: N6-methyladenosine modification of CENPK mRNA by ZC3H13 promotes cervical cancer stemness and chemoresistance
Source: Mil Med Res. 2022 Apr 14;9:19. doi: 10.1186/s40779-022-00378-z (PMC9008995; doi:10.1186/s40779-022-00378-z)
Supplement: Supplementary file 1 — Additional file 1. Table S1 Sequences used in this study. Table S2 A list of antibodies used for ChIP, Co-IP, IF, IHC, and WB. Table S3 Correlation between CENPK expression and the clinicopathological characteristics of cervical cancer patients. [file 40779_2022_378_MOESM1_ESM.pdf]

Table S1 Sequences used in this study

siRNAs			Sequences
CENPK siRNA	1	Sense	5’ GCUGCUGCGUAAUGGAAUU dTdT 3’
		Antisense	3’ dTdT CGACGACGCAUUACCUUAA 5’
	2	Sense	5’ GGAACAACGGUGGUUGGAU dTdT 3’
		Antisense	3’ dTdT CCUUGUUGCCACCAACCUA 5’
SOX6 siRNA	1	Sense	5’ CCACCCACAAAUUAACCAA dTdT 3’
		Antisense	3’ dTdT GGUGGGUGUUUAAUUGGUU 5’
	2	Sense	5’ GCCACUCUUACAACCACAA dTdT 3’
		Antisense	3’ dTdT CGGUGAGAAUGUUGGUGUU 5’
p53 siRNA	1	Sense	5’ GACUCCAGUGGUAAUCUAC dTdT 3’
		Antisense	3’ dTdT CUGAGGUCACCAUUAGAUG 5’
	2	Sense	5’ CUACUUCCUGAAAACAACG dTdT 3’
		Antisense	3’ dTdT GAUGAAGGACUUUUGUUGC 5’
ZC3H13 siRNA	1	Sense	5’ GAGAGAGCUCCUAUGUUCU dTdT 3’
		Antisense	3’ dTdT CUCUCUCGAGGAUACAAGA 5’
	2	Sense	5’ GCUCCUAUGUUCUACACCU dTdT 3’
		Antisense	3’ dTdT CGAGGAUACAAGAUGUGGA 5’
Negative control		Sense	5’ UUCUCCGAACGUGUCACGU dTdT 3’
		Antisense	3’ dTdT AAGAGGCUUGCACAGUGCA 5’

Table S2 A list of antibodies used for ChIP, Co-IP, IF, IHC, and WB

Antibody	Cat. No	Company	Species	Dulution
Flag	F7425	Sigma	Rabbit	1:1000 (WB); 1:20 (Co-IP)
CENPK	ab236739	Abcam	Rabbit	1:1000 (WB); 1:20 (Co-IP); 1:100 (IF); 1:100 (IHC)
ZC3H13	ab70802	Abcam	Rabbit	1:2000 (WB)
Ki67	ab16667	Abcam	Rabbit	1:100 (IHC)
SOX6	ab243576	Abcam	Mouse	1:100 (IF)
γ-H2AX	ab11174	Abcam	Rabbit	1:500 (IF)
SOX6	ab125196	Abcam	Rabbit	1:1000 (WB); 1:20 (Co-IP)
SOX6	PA5-34616	Invitrogen	Rabbit	1:50 (ChIP)
β-catenin	8480	CST	Rabbit	1:1000 (WB); 1:100 (IF)
c-Jun	19807	CST	Rabbit	1:1000 (WB)
CD44	3570	CST	Mouse	1:1000 (IF)
CD133	18470-1-AP	Proteintech	Rabbit	1:1000 (IF)
c-Myc	10828-1-AP	Proteintech	Rabbit	1:1000 (WB)
CCND1	60186-1-Ig	Proteintech	Mouse	1:1000 (WB)
p53	10442-1-AP	Proteintech	Rabbit	1:1000 (WB)
p21	10355-1-AP	Proteintech	Rabbit	1:1000 (WB)
Vimentin	10366-1-AP	Proteintech	Rabbit	1:1000 (WB)
Ubiquitin	10201-2-AP	Proteintech	Rabbit	1:1000 (WB)
GAPDH	60004-1-Ig	Proteintech	Mouse	1:5000 (WB)
Histone	17168-1-AP	Proteintech	Rabbit	1:1000 (WB)
β-actin	60008-1-Ig	Proteintech	Mouse	1:5000 (WB)

ChIP chromatin immunoprecipitation, *IF* immunofluorescence, *IHC* immunohistochemistry, *Co-IP* co-immunoprecipitation, *WB* western blotting

Table S3 Correlation between CENPK expression and the clinicopathological characteristics of cervical cancer patients

Characteristics	CENPK expression [n(%)]		P
	Low	High	
Age (years)			0.427
≤ Median (n = 58)	27 (46.6)	31 (53.4)	
> Median (n = 61)	24 (39.3)	37 (60.7)	
pT classification			0.384
T_1-T_2 (n = 96)	43 (44.8)	53 (55.2)	
T_3-T_4 (n = 23)	8 (34.8)	15 (65.2)	
pN classification			0.247
N_0 (n = 97)	44 (45.4)	53 (54.6)	
N_1 (n = 22)	7 (31.8)	15 (68.2)	
Distant metastasis			
No (n = 119)	51 (42.9)	68 (57.1)	
Yes (n = 0)	0 (0)	0 (0)	
Recurrence			0.008
No (n = 80)	41 (51.2)	39 (48.8)	
Yes (n = 39)	10 (25.6)	29 (74.4)	
Histology grade			0.670
I-II (n = 27)	12 (44.4)	15 (55.6)	
III (n = 73)	29 (39.7)	44 (60.3)	
Ki67 status			< 0.001
Low expression (n = 56)	33 (58.9)	23 (41.1)	
High expression (n = 41)	8 (19.5)	33 (80.5)	
