# Supplementary material for: N6-methyladenosine modification of CENPK mRNA by ZC3H13 promotes cervical cancer stemness and chemoresistance
Source: Mil Med Res. 2022 Apr 14;9:19. doi: 10.1186/s40779-022-00378-z (PMC9008995; doi:10.1186/s40779-022-00378-z)
Supplement: Supplementary file 2 — Additional file 2. Fig. S1 DNA copy number variation of 21 m6A regulators in cervical cancer based on TCGA database. Fig. S2 Genetic and expression variation profiles of m6A regulators in cervical cancer based on TCGA database. Fig. S3 Survival analysis of cervical patients in TCGA database. Fig. S4 Unsupervised clustering of m6A regulators as per TCGA CESC dataset. Fig. S5 Gene set variation analysis identifies the involvement of biological characteristics in distinct m6A methylation patterns. Fig. S6 Bioinformatics analyses of m6A-modified CENPK based on the microarray and TCGA data. Fig. S7 ZC3H13 augments pro-tumorigenic functions of cervical cancer cells through CENPK-modulated Wnt and p53 signaling. Fig. S8 CENPK confers poor patient prognosis by stratified analysis and its relationship with related genes expression. Fig. S9 CENPK promotes cervical cancer stemness, chemoresistance, metastasis, and proliferation. Fig. S10 CENPK augments pro-tumorigenic functions of cervical cancer cells through Wnt and p53 signaling. [file 40779_2022_378_MOESM2_ESM.pdf]

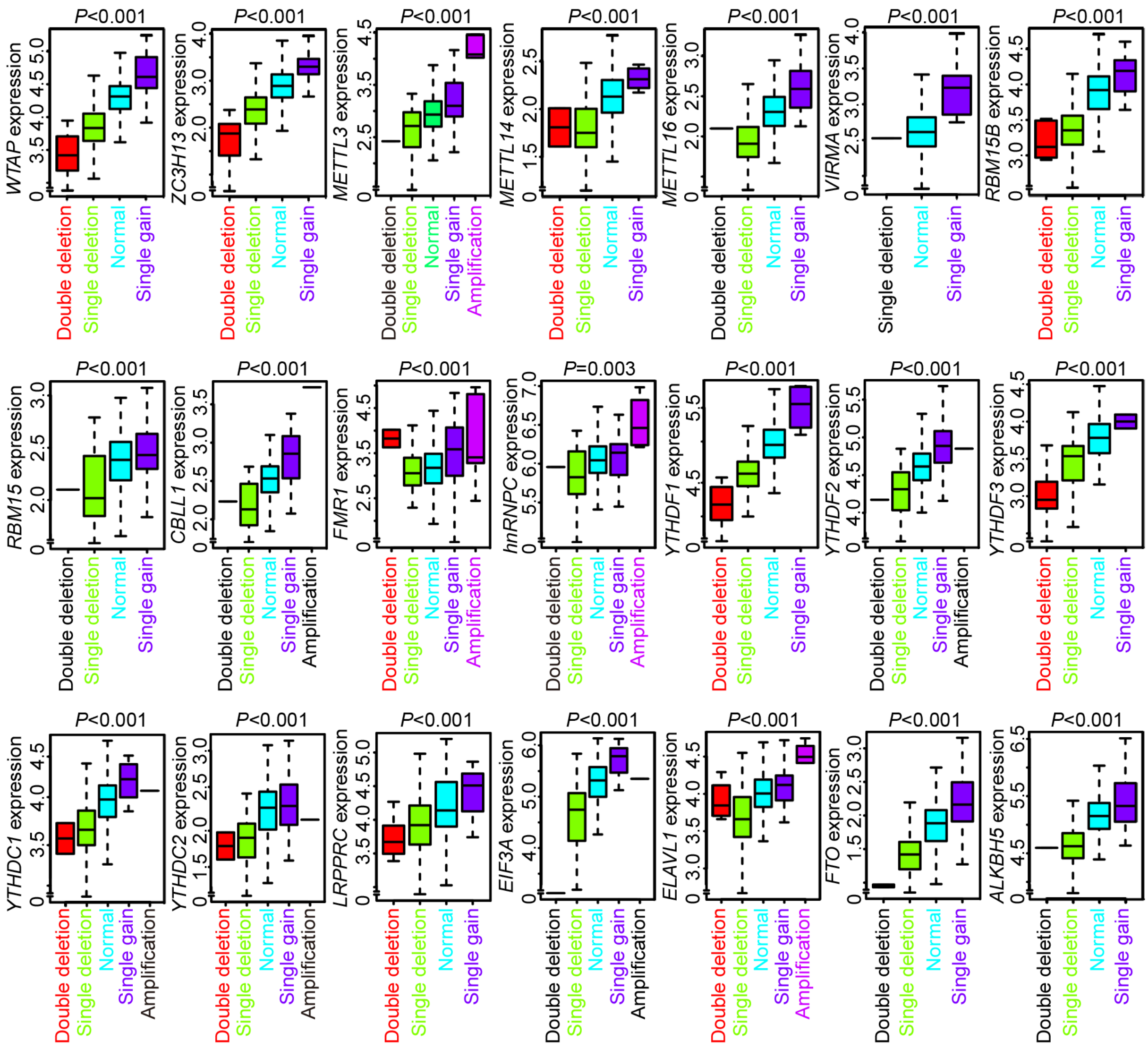

Fig. S1 DNA copy number variation of 21 m^{6}A regulators in cervical cancer based on TCGA database. Differential expression of 21 m^{6}A regulators [9 m^{6}A writers (including *WTAP*, *ZC3H13*, *METTL3*, *METTL14*, *METTL16*, *VIRMA*, *RBM15B*, *RBM15*, and *CBLL1*), 10 m^{6}A readers (including *FMR1*, *hnRNPC*, *YTHDF1/2/3*, *YTHDC1/2*, *LRPPRC*, *EIF3A*, and *ELAVL1*), and 2 m^{6}A erasers (*FTO* and *ALKBH5*)] in cervical cancer patients with gene copy number variations (CNV), including gene double deletion, single deletion, single gain, and amplification

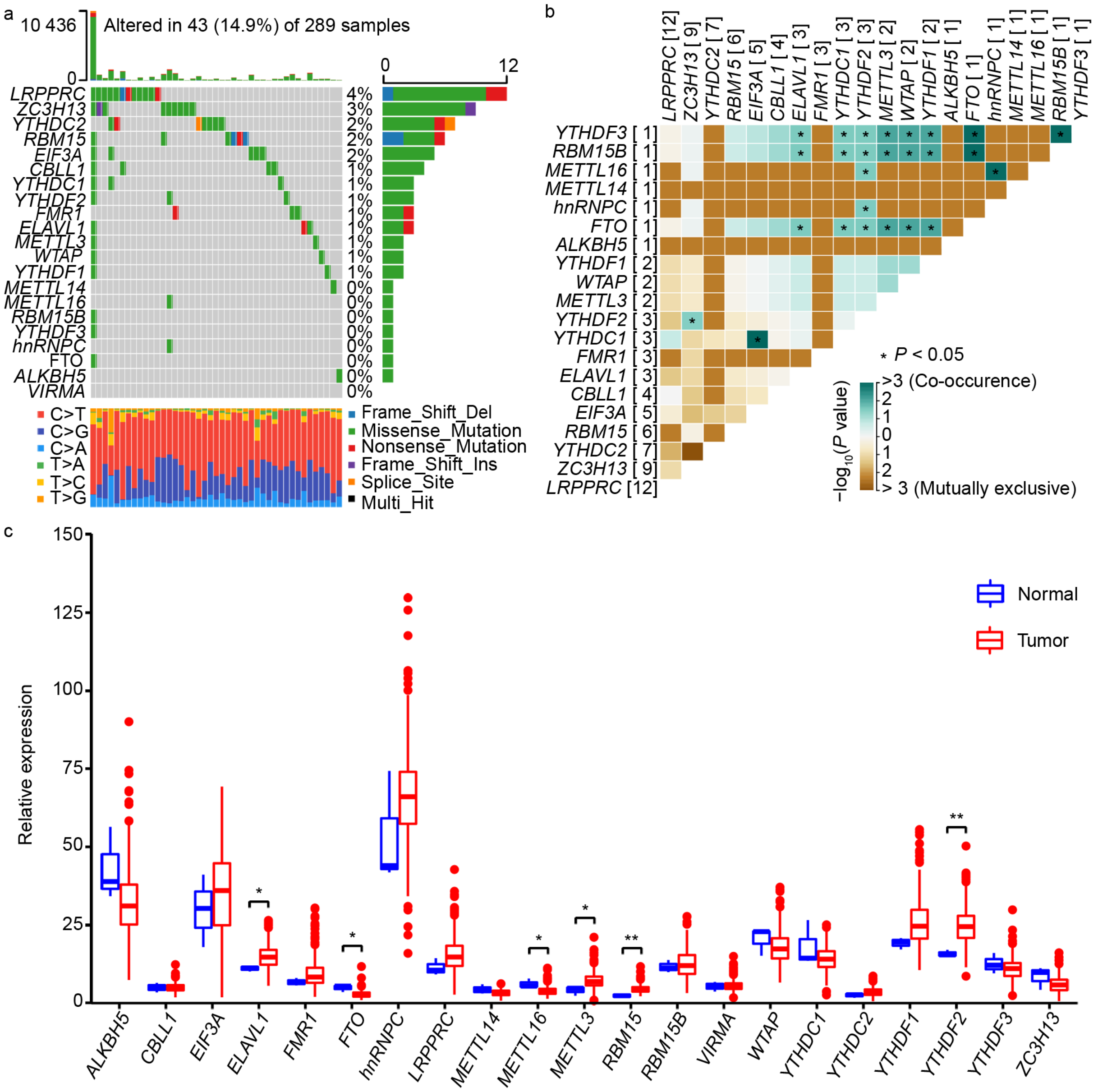

Fig. S2 Genetic and expression variation profiles of m^{6}A regulators in cervical cancer based on TCGA database. **a** Mutation frequency and mutation type of m^{6}A regulators in cervical cancer. **b** Co-occurrence and exclusive of the mutations for m^{6}A regulators. The numbers in square brackets beside each gene represented the number of samples with the specific gene mutation. **c** Expression of 21 m^{6}A regulators between cervical cancer (n = 307) and adjacent normal tissues (n = 3) (Wilcoxon rank-sum tests). $^{*}P < 0.05$, $^{**}P < 0.01$

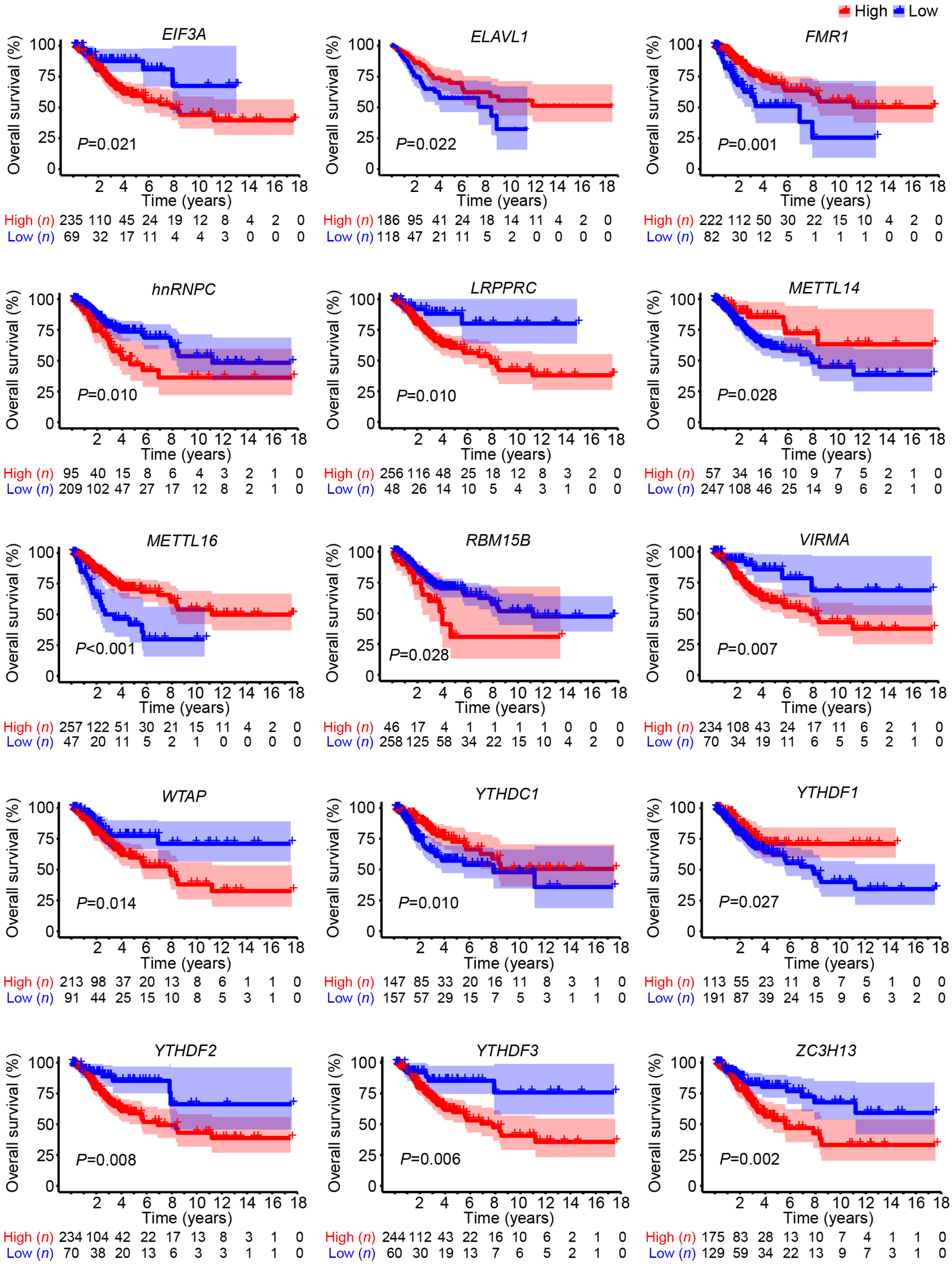

Fig. S3 Survival analysis of cervical patients in TCGA database. Kaplan-Meier curves of overall patient survival based on the expression of 15 m^{6}A regulators, including *EIF3A*, *ELAVL1*, *FMR1*, *hnRNPC*, *LRPPRC*, *METTL14*, *METTL16*, *RBM15B*, *VIRMA*, *WTAP*, *YTHDC1*, *YTHDF1*, *YTHDF2*, *YTHDF3*, and *ZC3H13*

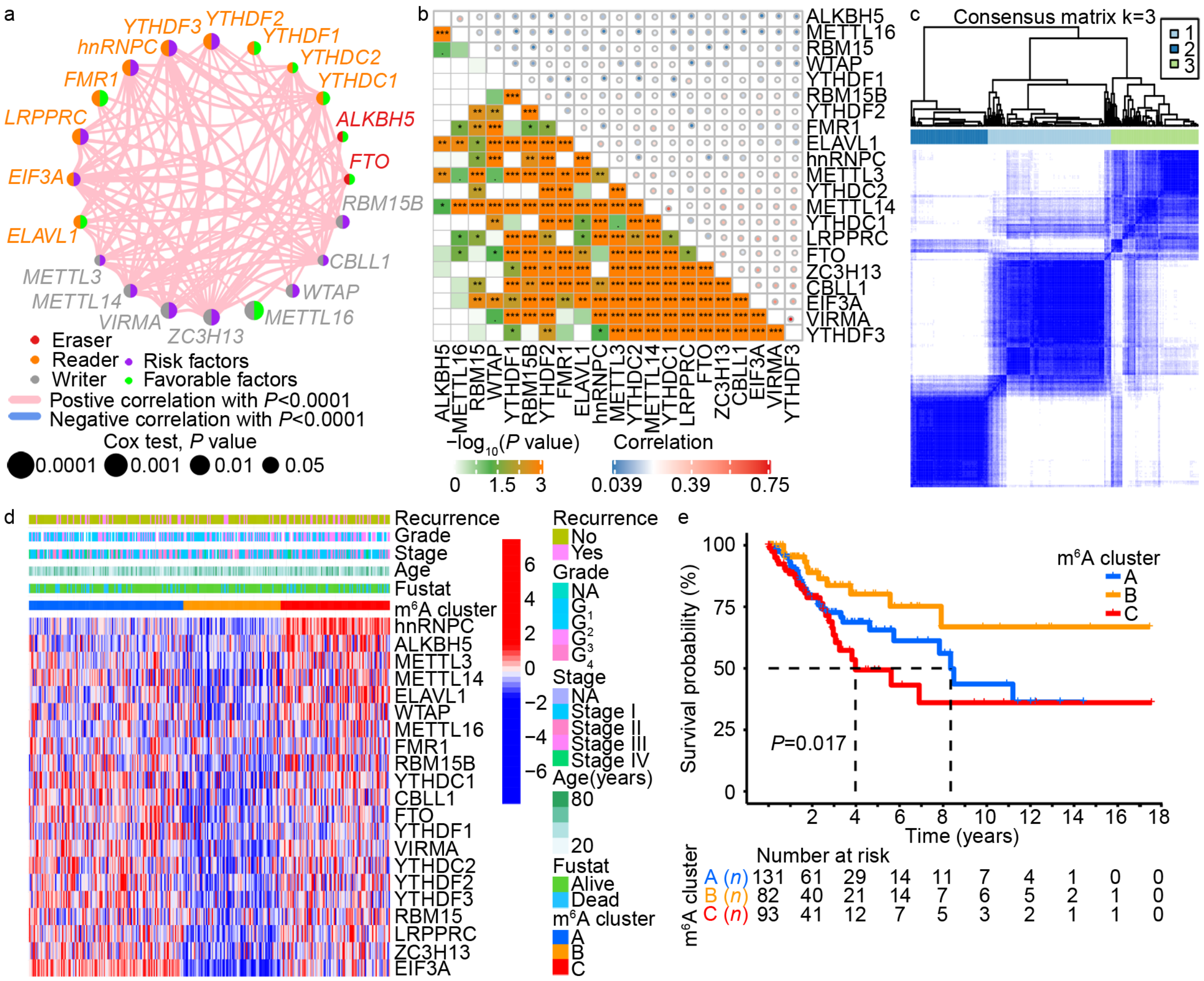

Fig. S4 Unsupervised clustering of m^{6}A regulators as per TCGA CESC dataset. **a** Interplay between 21 m^{6}A regulators and their prognostic value calculated by Log-rank test in cervical cancer. **b**Correlogram displaying the relationship among 21 m^{6}A regulators expression. **c** Consensus matrices of the TCGA CESC cohort for k = 3. **d** Distribution of 21 m^{6}A regulators in 3 m^{6}A modification patterns and samples from TCGA CESC cohort with distinct clinical characteristics. **e** Kaplan-Meier curves were plotted for survival analysis of 3 m^{6}A modification patterns

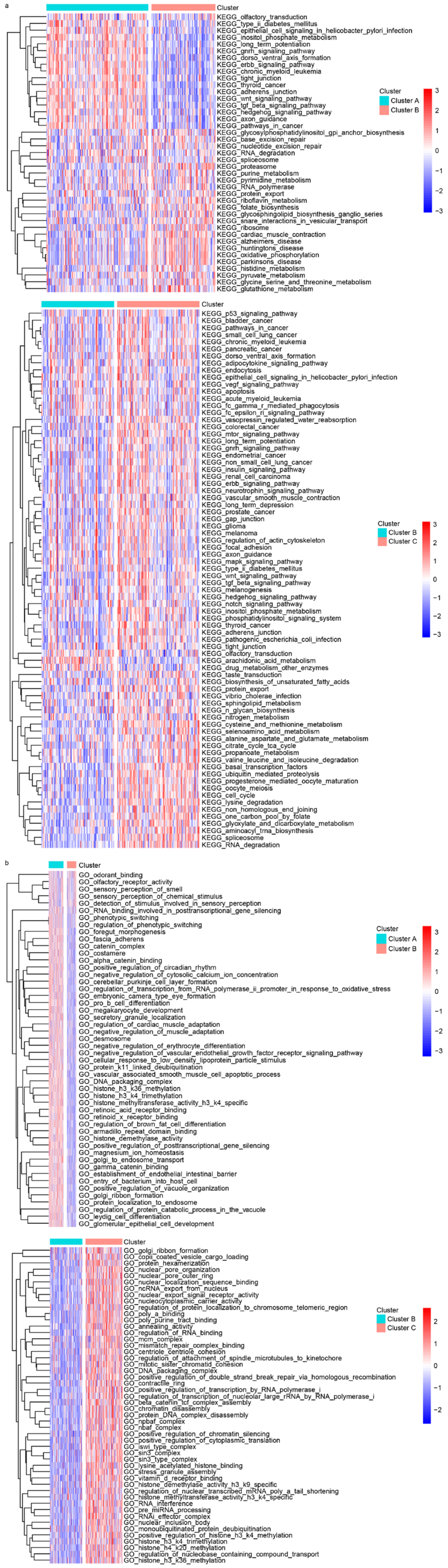

a
Cluster
KEGG_olfactory_transduction
KEGG_type_ii_diabetes_mellitus
KEGG_epithelial_cell_signaling_in_helicobacter_pylori_infection
KEGG_inositol_phosphate_metabolism
KEGG_long_term_potentiation
KEGG_gnrh_signaling_pathway
KEGG_dorso_ventral_axis_formation
KEGG_erbb_signaling_pathway
KEGG_chronic_myeloid_leukemia
KEGG_tight_junction
KEGG_thyroid_cancer
KEGG_adherens_junction
KEGG_wnt_signaling_pathway
KEGG_tgf_beta_signaling_pathway
KEGG_hedgehog_signaling_pathway
KEGG_axon_guidance
KEGG_pathways_in_cancer
KEGG_glycosylphosphatidylinositol_gpi_anchor_biosynthesis
KEGG_base_excision_repair
KEGG_nucleotide_excision_repair
KEGG_RNA_degradation
KEGG_spliceosome
KEGG_proteasome
KEGG_purine_metabolism
KEGG_pyrimidine_metabolism
KEGG_RNA_polymerase
KEGG_protein_export
KEGG_riboflavin_metabolism
KEGG_folate_biosynthesis
KEGG_glycosphingolipid_biosynthesis_ganglio_series
KEGG_snare_interactions_in_vesicular_transport
KEGG_ribosome
KEGG_cardiac_muscle_contraction
KEGG_alzheimers_disease
KEGG_huntingtons_disease
KEGG_oxidative_phosphorylation
KEGG_parkinsons_disease
KEGG_histidine_metabolism
KEGG_pyruvate_metabolism
KEGG_glycine_serine_and_threonine_metabolism
KEGG_glutathione_metabolism
Cluster
Cluster A
Cluster B
3
2
1
0
−1
−2
−3
Cluster
KEGG_p53_signaling_pathway
KEGG_bladder_cancer
KEGG_pathways_in_cancer
KEGG_small_cell_lung_cancer
KEGG_chronic_myeloid_leukemia
KEGG_pancreatic_cancer
KEGG_dorso_ventral_axis_formation
KEGG_adipocytokine_signaling_pathway
KEGG_endocytosis
KEGG_epithelial_cell_signaling_in_helicobacter_pylori_infection
KEGG_vegf_signaling_pathway
KEGG_apoptosis
KEGG_acute_myeloid_leukemia
KEGG_fc_gamma_r_mediated_phagocytosis
KEGG_fc_epsilon_ri_signaling_pathway
KEGG_vasopressin_regulated_water_reabsorption
KEGG_colorectal_cancer
KEGG_mtor_signaling_pathway
KEGG_long_term_potentiation
KEGG_gnrh_signaling_pathway
KEGG_endometrial_cancer
KEGG_non_small_cell_lung_cancer
KEGG_insulin_signaling_pathway
KEGG_renal_cell_carcinoma
KEGG_erbb_signaling_pathway
KEGG_neurotrophin_signaling_pathway
KEGG_vascular_smooth_muscle_contraction
KEGG_long_term_depression
KEGG_prostate_cancer
KEGG_gap_junction
KEGG_glioma
KEGG_melanoma
KEGG_regulation_of_actin_cytoskeleton
KEGG_focal_adhesion
KEGG_axon_guidance
KEGG_mapk_signaling_pathway
KEGG_type_ii_diabetes_mellitus
KEGG_wnt_signaling_pathway
KEGG_tgf_beta_signaling_pathway
KEGG_melanogenesis
KEGG_hedgehog_signaling_pathway
KEGG_notch_signaling_pathway
KEGG_inositol_phosphate_metabolism
KEGG_phosphatidylinositol_signaling_system
KEGG_thyroid_cancer
KEGG_adherens_junction
KEGG_pathogenic_escherichia_coli_infection
KEGG_tight_junction
KEGG_olfactory_transduction
KEGG_arachidonic_acid_metabolism
KEGG_drug_metabolism_other_enzymes
KEGG_taste_transduction
KEGG_biosynthesis_of_unsaturated_fatty_acids
KEGG_protein_export
KEGG_vibrio_cholerae_infection
KEGG_sphingolipid_metabolism
KEGG_n_glycan_biosynthesis
KEGG_nitrogen_metabolism
KEGG_cysteine_and_methionine_metabolism
KEGG_selenoamino_acid_metabolism
KEGG_alanine_aspartate_and_glutamate_metabolism
KEGG_citrate_cycle_tca_cycle
KEGG_propanoate_metabolism
KEGG_valine_leucine_and_isoleucine_degradation
KEGG_basal_transcription_factors
KEGG_ubiquitin_mediated_proteolysis
KEGG_progesterone_mediated_oocyte_maturation
KEGG_oocyte_meiosis
KEGG_cell_cycle
KEGG_lysine_degradation
KEGG_non_homologous_end_joining
KEGG_one_carbon_pool_by_folate
KEGG_glyoxylate_and_dicarboxylate_metabolism
KEGG_aminoacyl_trna_biosynthesis
KEGG_spliceosome
KEGG_RNA_degradation
Cluster
Cluster B
Cluster C
3
2
1
0
−1
−2
−3
b
Cluster
GO_odorant_binding
GO_olfactory_receptor_activity
GO_sensory_perception_of_smell
GO_sensory_perception_of_chemical_stimulus
GO_detection_of_stimulus_involved_in_sensory_perception
GO_RNA_binding_involved_in_posttranscriptional_gene_silencing
GO_phenotypic_switching
GO_regulation_of_phenotypic_switching
GO_foregut_morphogenesis
GO_fascia_adherens
GO_catenin_complex
GO_costamere
GO_alpha_catenin_binding
GO_positive_regulation_of_circadian_rhythm
GO_negative_regulation_of_cytosolic_calcium_ion_concentration
GO_cerebellar_purkinje_cell_layer_formation
GO_regulation_of_transcription_from_RNA_polymerase_ii_promoter_in_response_to_oxidative_stress
GO_embryonic_camera_type_eye_formation
GO_pro_b_cell_differentiation
GO_megakaryocyte_development
GO_secretory_granule_localization
GO_regulation_of_cardiac_muscle_adaptation
GO_negative_regulation_of_muscle_adaptation
GO_desmosome
GO_negative_regulation_of_erythrocyte_differentiation
GO_negative_regulation_of_vascular_endothelial_growth_factor_receptor_signaling_pathway
GO_cellular_response_to_low_density_lipoprotein_particle_stimulus
GO_protein_k11_linked_deubiquitination
GO_vascular_associated_smooth_muscle_cell_apoptotic_process
GO_DNA_packaging_complex
GO_histone_h3_k36_methylation
GO_histone_h3_k4_trimethylation
GO_histone_methyltransferase_activity_h3_k4_specific
GO_retinoic_acid_receptor_binding
GO_retinoid_x_receptor_binding
GO_regulation_of_brown_fat_cell_differentiation
GO_armadillo_repeat_domain_binding
GO_histone_demethylase_activity
GO_positive_regulation_of_posttranscriptional_gene_silencing
GO_magnesium_ion_homeostasis
GO_golgi_to_endosome_transport
GO_gamma_catenin_binding
GO_establishment_of_endothelial_intestinal_barrier
GO_entry_of_bacterium_into_host_cell
GO_positive_regulation_of_vacuole_organization
GO_golgi_ribbon_formation
GO_protein_localization_to_endosome
GO_regulation_of_protein_catabolic_process_in_the_vacuole
GO_leydig_cell_differentiation
GO_glomerular_epithelial_cell_development
Cluster
Cluster A
Cluster B
3
2
1
0
−1
−2
−3
Cluster
GO_golgi_ribbon_formation
GO_copii_coated_vesicle_cargo_loading
GO_protein_hexamerization
GO_nuclear_pore_organization
GO_nuclear_pore_outer_ring
GO_nuclear_localization_sequence_binding
GO_ncRNA_export_from_nucleus
GO_nuclear_export_signal_receptor_activity
GO_nucleocytoplasmic_carrier_activity
GO_regulation_of_protein_localization_to_chromosome_telomeric_region
GO_poly_a_binding
GO_poly_purine_tract_binding
GO_annealing_activity
GO_regulation_of_RNA_binding
GO_mcm_complex
GO_mismatch_repair_complex_binding
GO_centriole_centriole_cohesion
GO_regulation_of_attachment_of_spindle_microtubules_to_kinetochore
GO_mitotic_sister_chromatid_cohesion
GO_DNA_packaging_complex
GO_positive_regulation_of_double_strand_break_repair_via_homologous_recombination
GO_contractile_ring
GO_positive_regulation_of_transcription_by_RNA_polymerase_i
GO_regulation_of_transcription_of_nucleolar_large_rRNA_by_RNA_polymerase_i
GO_beta_catenin_tcf_complex_assembly
GO_chromatin_disassembly
GO_protein_DNA_complex_disassembly
GO_npbaf_complex
GO_nbaf_complex
GO_positive_regulation_of_chromatin_silencing
GO_positive_regulation_of_cytoplasmic_translation
GO_iswi_type_complex
GO_sin3_complex
GO_sin3_type_complex
GO_lysine_acetylated_histone_binding
GO_stress_granule_assembly
GO_vitamin_d_receptor_binding
GO_histone_demethylase_activity_h3_k9_specific
GO_regulation_of_nuclear_transcribed_mRNA_poly_a_tail_shortening
GO_histone_methyltransferase_activity_h3_k4_specific
GO_RNA_interference
GO_pre_miRNA_processing
GO_RNAi_effector_complex
GO_nuclear_inclusion_body
GO_monoubiquitinated_protein_deubiquitination
GO_positive_regulation_of_histone_h3_k4_methylation
GO_histone_h3_k4_trimethylation
GO_histone_h4_k20_methylation
GO_regulation_of_nucleobase_containing_compound_transport
GO_histone_h3_k36_methylation
Cluster
Cluster B
Cluster C
2
1
0
−1
−2

Fig. S5 Gene set variation analysis identifies the involvement of biological characteristics in distinct m^{6}A methylation patterns. Heatmaps showing the enriched KEGG (**a**) and GO (**b**) sets among distinct m^{6}A methylation patterns as per TCGA CESC dataset

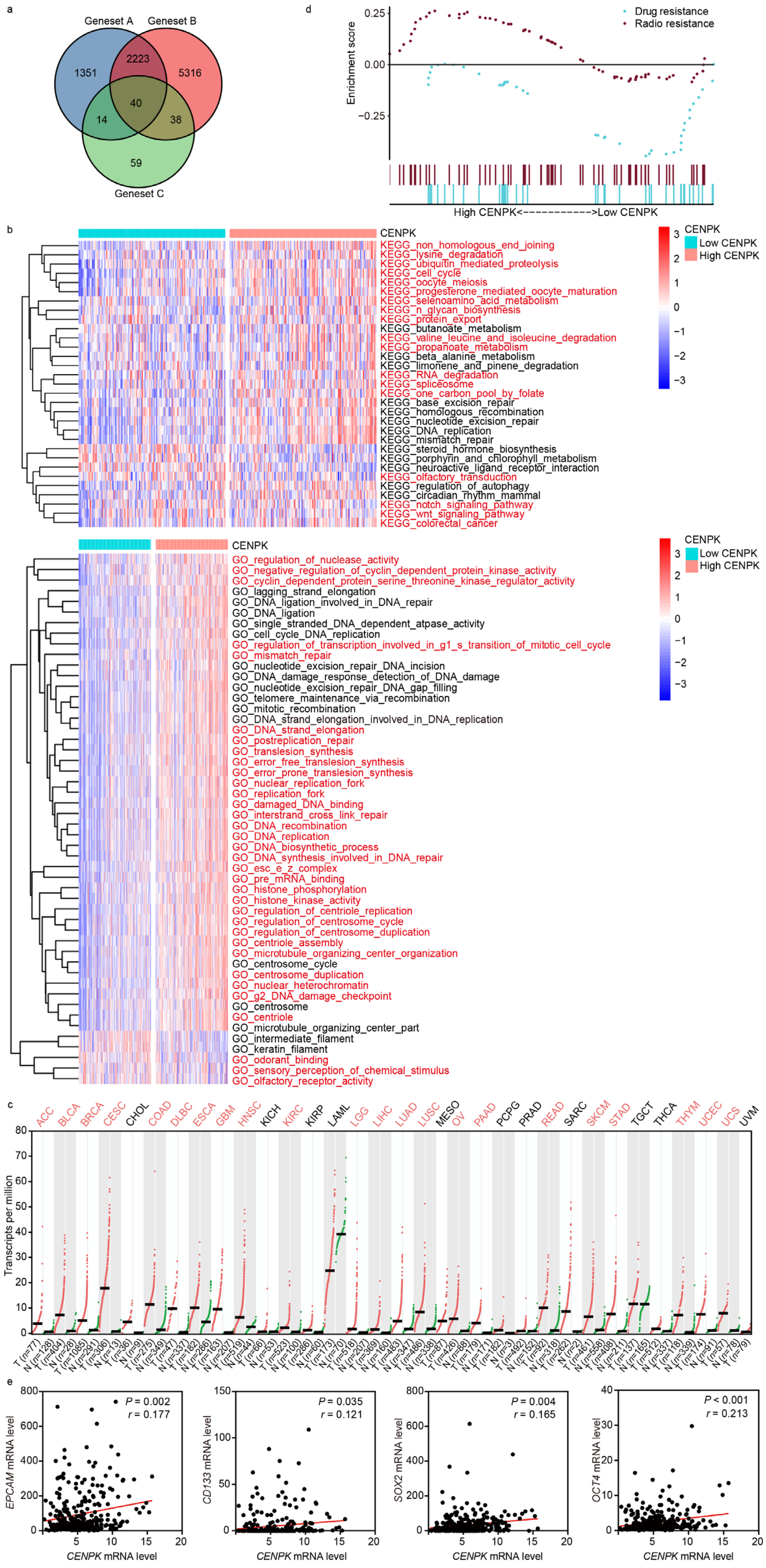
a
Geneset A
Geneset B
Geneset C
1351
2223
5316
40
14
38
59
d
Drug resistance
Radio resistance
Enrichment score
0.25
0.00
−0.25
High CENPK<----------->Low CENPK
b
CENPK
Low CENPK
High CENPK
3
2
1
0
−1
−2
−3
KEGG_non_homologous_end_joining
KEGG_lysine_degradation
KEGG_ubiquitin_mediated_proteolysis
KEGG_cell_cycle
KEGG_oocyte_meiosis
KEGG_progesterone_mediated_oocyte_maturation
KEGG_selenoamino_acid_metabolism
KEGG_n_glycan_biosynthesis
KEGG_protein_export
KEGG_butanoate_metabolism
KEGG_valine_leucine_and_isoleucine_degradation
KEGG_propanoate_metabolism
KEGG_beta_alanine_metabolism
KEGG_limonene_and_pinene_degradation
KEGG_RNA_degradation
KEGG_spliceosome
KEGG_one_carbon_pool_by_folate
KEGG_base_excision_repair
KEGG_homologous_recombination
KEGG_nucleotide_excision_repair
KEGG_DNA_replication
KEGG_mismatch_repair
KEGG_steroid_hormone_biosynthesis
KEGG_porphyrin_and_chlorophyll_metabolism
KEGG_neuroactive_ligand_receptor_interaction
KEGG_olfactory_transduction
KEGG_regulation_of_autophagy
KEGG_circadian_rhythm_mammal
KEGG_notch_signaling_pathway
KEGG_wnt_signaling_pathway
KEGG_colorectal_cancer
GO_regulation_of_nuclease_activity
GO_negative_regulation_of_cyclin_dependent_protein_kinase_activity
GO_cyclin_dependent_protein_serine_threonine_kinase_regulator_activity
GO_lagging_strand_elongation
GO_DNA_ligation_involved_in_DNA_repair
GO_DNA_ligation
GO_single_stranded_DNA_dependent_atpase_activity
GO_cell_cycle_DNA_replication
GO_regulation_of_transcription_involved_in_g1_s_transition_of_mitotic_cell_cycle
GO_mismatch_repair
GO_nucleotide_excision_repair_DNA_incision
GO_DNA_damage_response_detection_of_DNA_damage
GO_nucleotide_excision_repair_DNA_gap_filling
GO_telomere_maintenance_via_recombination
GO_mitotic_recombination
GO_DNA_strand_elongation_involved_in_DNA_replication
GO_DNA_strand_elongation
GO_postreplication_repair
GO_translesion_synthesis
GO_error_free_translesion_synthesis
GO_error_prone_translesion_synthesis
GO_nuclear_replication_fork
GO_replication_fork
GO_damaged_DNA_binding
GO_interstrand_cross_link_repair
GO_DNA_recombination
GO_DNA_replication
GO_DNA_biosynthetic_process
GO_DNA_synthesis_involved_in_DNA_repair
GO_esc_e_z_complex
GO_pre_mRNA_binding
GO_histone_phosphorylation
GO_histone_kinase_activity
GO_regulation_of_centriole_replication
GO_regulation_of_centrosome_cycle
GO_regulation_of_centrosome_duplication
GO_centriole_assembly
GO_microtubule_organizing_center_organization
GO_centrosome_cycle
GO_centrosome_duplication
GO_nuclear_heterochromatin
GO_g2_DNA_damage_checkpoint
GO_centrosome
GO_centriole
GO_microtubule_organizing_center_part
GO_intermediate_filament
GO_keratin_filament
GO_odorant_binding
GO_sensory_perception_of_chemical_stimulus
GO_olfactory_receptor_activity
c
ACC BLCA BRCA CESC CHOL COAD DLBC ESCA GBM HNSC KICH KIRC KIRP LAML LGG LIHC LUAD LUSC MESO OV PAAD PCPG PRAD READ SARC SKCM STAD TGCT THCA THYM UCEC UCS UVM
Transcripts per million
0
10
20
30
40
50
60
70
80
e
EPCAM mRNA level
P = 0.002
r = 0.177
CD133 mRNA level
P = 0.035
r = 0.121
SOX2 mRNA level
P = 0.004
r = 0.165
OCT4 mRNA level
P < 0.001
r = 0.213
CENPK mRNA level

Fig. S6 Bioinformatics analyses of m^6A-modified *CENPK* based on the microarray and TCGA data. **a** A Venn diagram analysis for searching the potential downstream effectors functioned in cervical cancer progression and modified by m^6A RNA methylation based on the microarray and TCGA data. **b** Heatmaps of the differential enrichment of the gene sets that participated in regulating the Wnt, cell cycle, DNA replication, and DNA damage repair signaling between high and low *CENPK* expression based on TCGA database. Text in red color indicated the overlay of GSVA between cluster B *vs.* C and *CENPK* high *vs.* Low. **c** Comparison of *CENPK* expression between pan-cancer and adjacent normal tissues based on TCGA database. **d** GSEA of *CENPK* showing enrichment of the gene sets that participated in regulating platinum resistance and radioresistance based on TCGA database. **e** Relationships among *EPCAM*, *CD133*, *SOX2*, *OCT4*, and *CENPK* expression based on TCGA database

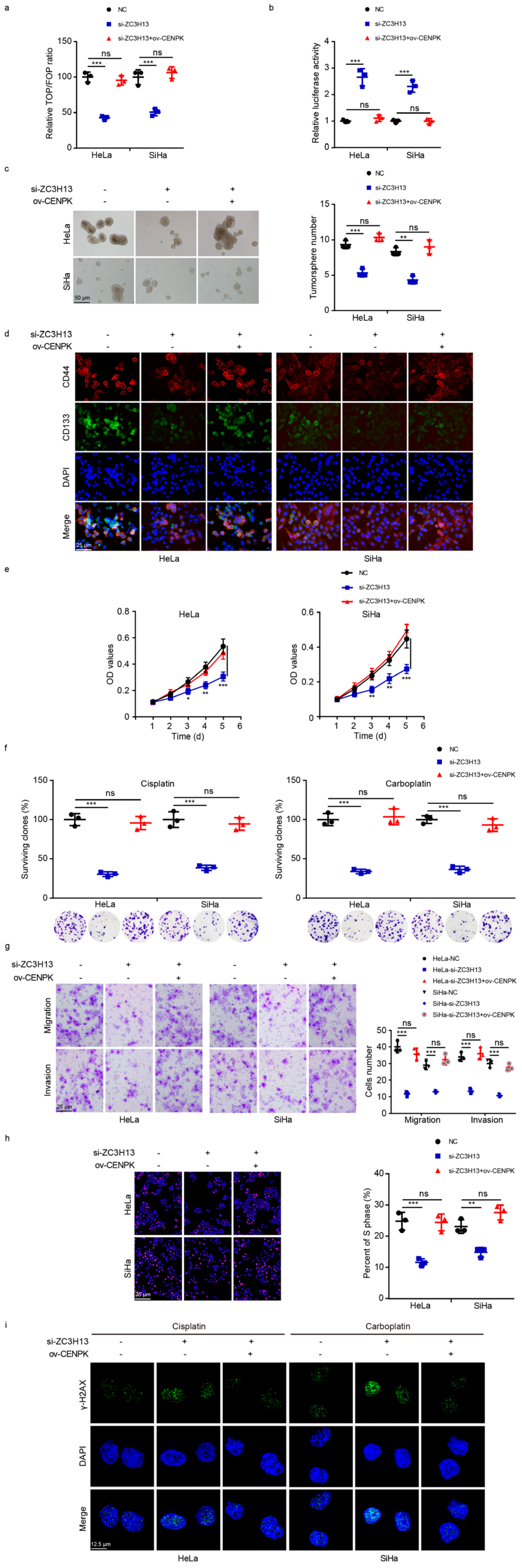
a
NC
si-ZC3H13
si-ZC3H13+ov-CENPK
Relative TOP/FOP ratio
HeLa
SiHa
b
Relative luciferase activity
c
si-ZC3H13
ov-CENPK
50 μm
Tumorsphere number
d
CD44
CD133
DAPI
Merge
25 μm
e
HeLa
SiHa
OD values
Time (d)
f
Cisplatin
Carboplatin
Surviving clones (%)
g
Migration
Invasion
HeLa-NC
HeLa-si-ZC3H13
HeLa-si-ZC3H13+ov-CENPK
SiHa-NC
SiHa-si-ZC3H13
SiHa-si-ZC3H13+ov-CENPK
Cells number
h
Percent of S phase (%)
20 μm
i
γ-H2AX
12.5 μm

Fig. S7 ZC3H13 augments pro-tumorigenic functions of cervical cancer cells through CENPK-modulated Wnt and p53 signaling. **a** TOP/FOP luciferase reporter assays were carried out for clarifying the effect of ZC3H13 and CENPK on Wnt signaling activity. **b** Luciferase reporter assays were adopted for elucidating the impact of ZC3H13 and CENPK on p53 signaling activity. Tumorsphere formation assays (**c**), immunofluorescence assays (**d**), MTT assays (**e**), clonogenic assays (**f**), transwell assays (**g**), and EdU incorporation assays (**h**) were applied for elucidating stemness, chemoresistance, metastasis, and proliferation of *ZC3H13*-depleted HeLa and SiHa cells, *ZC3H13*-depleted HeLa and SiHa cells with *CENPK* overexpression, and the control cells. **i** Immunofluorescence was adopted for detecting the expression of γ-H2AX (Ser139) in *ZC3H13*-depleted HeLa and SiHa cells, *ZC3H13*-depleted HeLa and SiHa cells with *CENPK* overexpression, and control cells following cisplatin (10 μmol/L for 24 h) or carboplatin (100 μmol/L for 24 h) treatment. Data are represented as mean ± SD. $^{**}P < 0.01$, $^{***}P < 0.001$; ns: non-significant

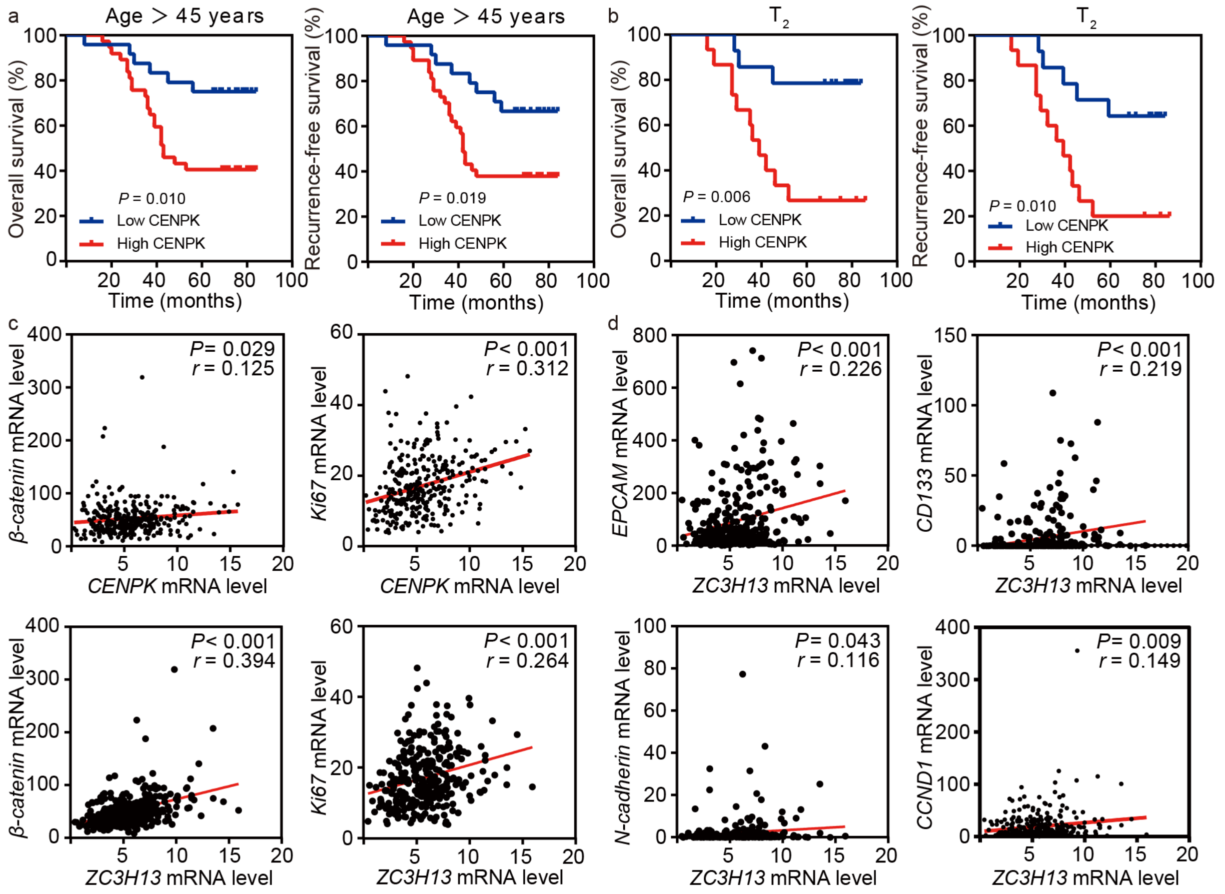

Fig. S8 CENPK confers poor patient prognosis by stratified analysis and its relationship with related genes expression. **a** Overall survival analysis and recurrence-free survival analysis of cervical cancer patients (age > 45 years old) based on CENPK expression. **b** Overall survival analysis and recurrence-free survival analysis of cervical cancer patients (T_2) based on CENPK expression. **c** Relationships among *β-catenin*, *Ki67*, and *CENPK* mRNA expression in TCGA database. **d** Relationships between *EPCAM*, *CD133*, *β-catenin*, *Ki67*, *N-cadherin*, *CCND1* and *CENPK* mRNA expression in TCGA database

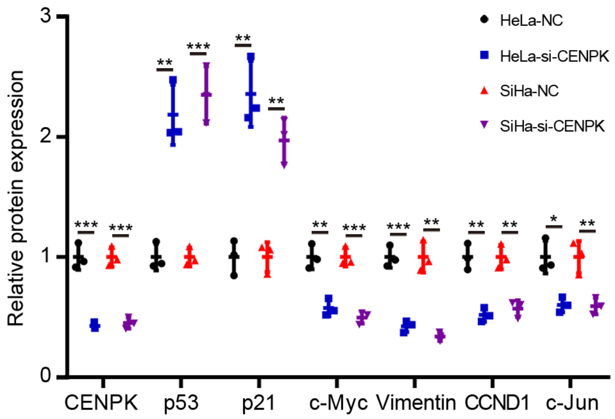

Fig. S9 CENPK promotes cervical cancer stemness, chemoresistance, metastasis, and proliferation. The quantification data of Western blotting detecting the expression of proteins associated with stemness (c-Myc), DNA damage repair (p53), epithelial-mesenchymal transition (Vimentin), and DNA replication (p21, CCND1 and c-Jun) in CENPK-depleted HeLa and SiHa cells and control cells. Data are represented as mean ± SD. $^{*}P < 0.05$, $^{**}P < 0.01$, $^{***}P < 0.001$

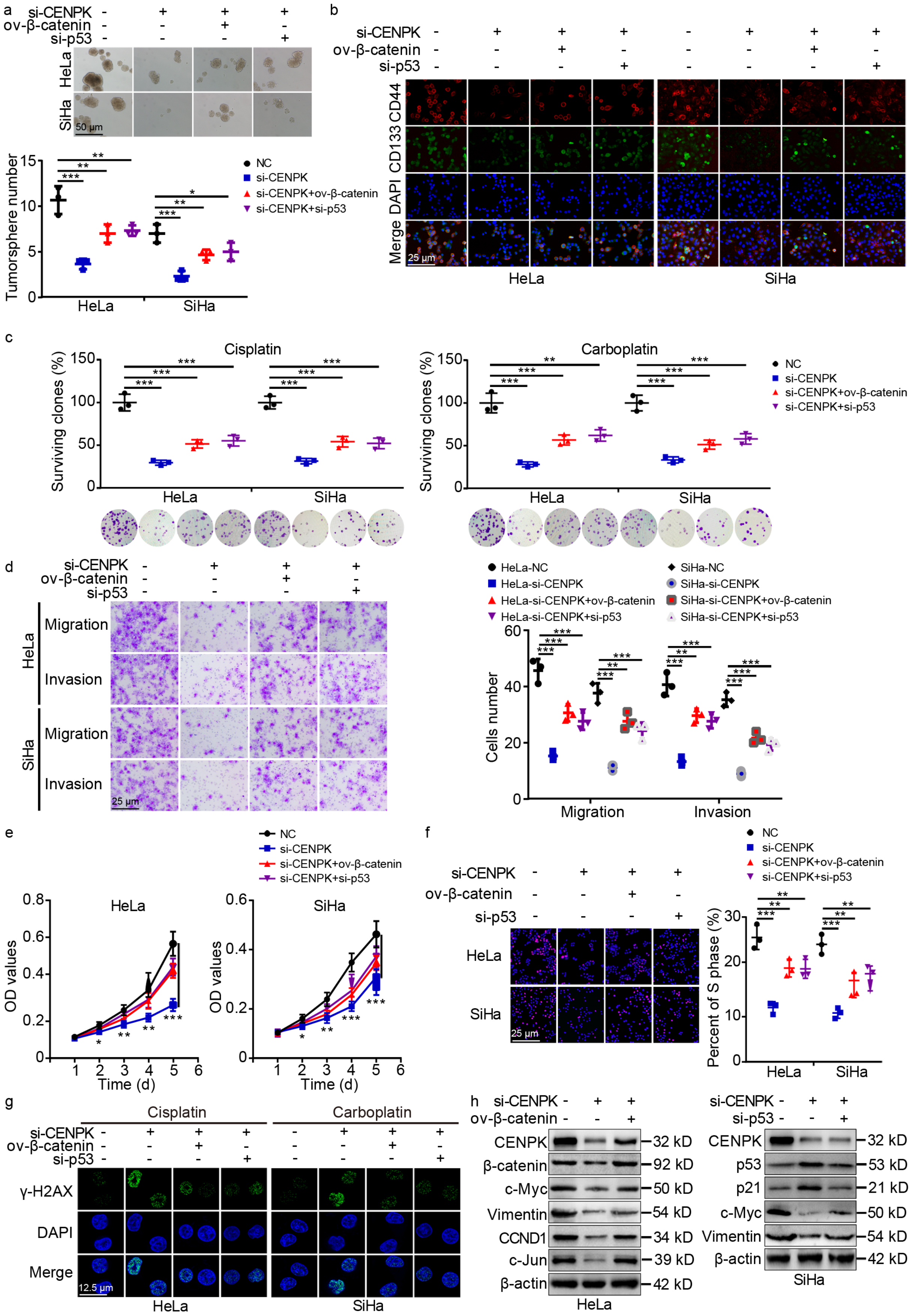
a
si-CENPK
ov-β-catenin
si-p53
HeLa
SiHa
50 μm
Tumorsphere number
NC
si-CENPK
si-CENPK+ov-β-catenin
si-CENPK+si-p53
b
CD44
CD133
DAPI
Merge
25 μm
c
Cisplatin
Carboplatin
Surviving clones (%)
d
Migration
Invasion
HeLa-NC
HeLa-si-CENPK
HeLa-si-CENPK+ov-β-catenin
HeLa-si-CENPK+si-p53
SiHa-NC
SiHa-si-CENPK
SiHa-si-CENPK+ov-β-catenin
SiHa-si-CENPK+si-p53
Cells number
e
OD values
Time (d)
f
Percent of S phase (%)
g
γ-H2AX
DAPI
Merge
12.5 μm
h
CENPK
β-catenin
c-Myc
Vimentin
CCND1
c-Jun
β-actin
p53
p21
32 kD
92 kD
50 kD
54 kD
34 kD
39 kD
42 kD
53 kD
21 kD

Fig. S10 CENPK augments pro-tumorigenic functions of cervical cancer cells through Wnt and p53 signaling. Tumorsphere formation assays (**a**), immunofluorescence assays (**b**), clonogenic assays (**c**), transwell assays (**d**), MTT assays (**e**), and EdU incorporation assays (**f**) were applied for evaluating stemness, chemoresistance, metastasis, and proliferation of *CENPK*-silenced HeLa and SiHa cells, *CENPK*-silenced HeLa and SiHa cells with *β-catenin* overexpression, *CENPK*-silenced HeLa and SiHa cells with *p53* knockdown, and the control cells. **g** Immunofluorescence was applied for detecting the expression of γ-H2AX (Ser139) in *CENPK*-silenced HeLa and SiHa cells, *CENPK*-silenced HeLa and SiHa cells with *β-catenin* overexpression, *CENPK*-silenced HeLa and SiHa cells with *p53* knockdown, and the control cells following cisplatin (10 μmol/L for 24 h) or carboplatin (100 μmol/L for 24 h) treatment. **h** Western blotting analysis of the expression of proteins associated with stemness (c-Myc), DNA damage repair (p53), epithelial-mesenchymal transition (Vimentin), and DNA replication (p21, CCND1 and c-Jun) in *CENPK*-silenced HeLa and SiHa cells, *CENPK*-silenced HeLa cells with *β-catenin* overexpression, *CENPK*-silenced SiHa cells with *p53* knockdown, and the control cells. Data are represented as mean ± SD. $^{*}P < 0.05$, $^{**}P < 0.01$, $^{***}P < 0.001$
